# Betacyanin Biosynthetic Genes and Enzymes Are Differentially Induced by (a)biotic Stress in *Amaranthus hypochondriacus*

**DOI:** 10.1371/journal.pone.0099012

**Published:** 2014-06-04

**Authors:** Gabriela Casique-Arroyo, Norma Martínez-Gallardo, Luis González de la Vara, John P. Délano-Frier

**Affiliations:** Centro de Investigación y de Estudios Avanzados-Unidad Irapuato, Irapuato, Guanajuato, México; National Taiwan University, Taiwan

## Abstract

An analysis of key genes and enzymes of the betacyanin biosynthetic pathway in *Amaranthus hypochondriacus* (*Ah*) was performed. Complete cDNA sequence of *Ah* genes coding for cyclo-DOPA 5-O glucosyltransferase (*AhcDOPA5-GT*), two 4, 5-DOPA-extradiol-dioxygenase isoforms (*AhDODA-1* and *AhDODA-2*, respectively), and a betanidin 5-*O*-glucosyltransferase (*AhB5-GT*), plus the partial sequence of an orthologue of the cytochrome P-450 *R* gene (*CYP76AD1*) were obtained. With the exception *AhDODA-2*, which had a closer phylogenetic relationship to *DODA-like* genes in anthocyanin-synthesizing plants, all genes analyzed closely resembled those reported in related Caryophyllales species. The measurement of basal gene expression levels, in addition to the DOPA oxidase tyrosinase (DOT) activity, in different tissues of three *Ah* genotypes having contrasting pigmentation levels (green to red-purple) was determined. Additional analyses were performed in *Ah* plants subjected to salt and drought stress and to two different insect herbivory regimes. Basal pigmentation accumulation in leaves, stems and roots of betacyanic plants correlated with higher expression levels of *AhDODA-1* and *AhB5-GT*, whereas DOT activity levels coincided with pigment accumulation in stems and roots and with the acyanic nature of green plants, respectively, but not with pigmentation in leaves. Although the abiotic stress treatments tested produced changes in pigment levels in different tissues, pigment accumulation was the highest in leaves and stems of drought stressed betacyanic plants, respectively. However, tissue pigment accumulation in stressed *Ah* plants did not always correlate with betacyanin biosynthetic gene expression levels and/or DOT activity. This effect was tissue- and genotype-dependent, and further suggested that other unexamined factors were influencing pigment content in stressed *Ah*. The results obtained from the insect herbivory assays, particularly in acyanic plants, also support the proposal that these genes could have functions other than betacyanin biosynthesis.

## Introduction

*Amaranthus hypochondriacus*, a grain amaranth, is a C4 dicot plant noted for its ability to tolerate stressful conditions and produce highly nutritious and health promoting seeds [1], [2]. Grain amaranths are appreciated for their capacity to withstand drought stress and salinity in soils due to their remarkable water use efficiency, higher than many other C3 and C4 crops [3]–[6]. Their drought-tolerance is also attributed to the physiological advantages conferred by the C4 pathway, an indeterminate flowering habit, growth of long taproots and extensive lateral root systems in response to water shortage in the soil, osmolyte accumulation and the expression of genes coding for scavengers of reactive oxygen species (ROS), protein stabilizers and transcription factors [7]–[12].

*A. hypochondriacus* is one of the approximately 70 species that comprise the genus *Amaranthus*, classified within the Amaranthaceae family, one of the 13 betalain producing families within the core Caryophyllales [13], [14]. Betalains are water-soluble, nitrogen-containing pigments with chemo-taxonomical value since, for still unresolved reasons, they have never been found jointly with anthocyanins in the same plant [13], [15]. They comprise the red-violet betacyanins and the yellow betaxanthins. Both are immonium conjugates of betalamic acid covalently bonded with cyclo-dihydroxyphenylalanine (cDOPA) glucosides (which can be further acylated, mostly with aromatic cinnamic acids) and amino acids or amines, respectively [16]–[18]. Studies performed with several genotypes and species of the genus *Amaranthus* determined that their pigmentation was due predominantly to two betacyanins: amaranthine, the most abundant form, and isoamaranthine. They are 5-*O*-glucuroindoglucosides of two aglycons: betanidin and isobetanidin (its C-15 epimer), respectively, which are known to accumulate at different ratios, depending on the species [16], [19]–[22].

The hydroxylation of tyrosine to dihydroxyphenylalanine (DOPA) and its further oxidation to DOPA quinone, both catalyzed by tyrosinases, are considered the first steps in the proposed biosynthetic pathway of betalains. Tyrosinases are copper-containing PPO-type bifunctional enzymes, proposed to catalyze the hydroxylation of phenols to o-diphenols (monophenol: monooxygenase) and their subsequent oxidation to o-quinones (o-diphenol: oxygen oxidoreductase) [23]. The involvement of tyrosinase activity in betalain biosynthesis is supported by extensive experimental evidence [24]–[31]. More recently, a cytochrome P450 gene was found to perform the biosynthetic step that provides the cyclo-DOPA moiety of red betacyanins in beet [32], whereas the identity of the gene coding for the tyrosinase-like enzyme catalyzing the conversion of tyrosine to L-3, 4-dihydroxyphenylalanine (L-DOPA) remains elusive. DOPA is then converted to betalamic acid, the chromophore molecule of both betacyanins and betaxanthins, by means of a crucial reaction catalyzed by a ring-opening extradiol DOPA-4, 5-dioxygenase (DODA). Based on evidence garnered from feeding experiments [33], [34], betalamic acid is believed to spontaneously condense with cDOPA, synthesized from DOPA by the action of the cytochrome P450 cDOPA synthase [27], [32], to produce betanidin. The subsequent transformation of betanidin to betanin is proposed to occur by two independent biosynthetic routes [14], [18]. In one, the glycosylation step leading to betanin is performed directly on betanidin by UDPG-dependent betanidin 5-O-glucosyltransferases, while in the other, betanin is produced after the condensation of betanidin with glucosylated cDOPA produced previously by the action of a cDOPA 5-O- glucosyltransferase. However, controversy still rages regarding which pathway is the main route for producing betacyanins *in vivo*, although extensive biochemical and molecular evidence in favor of the latter has been reported in several betacyanin-producing species [35]–[38], including the amaranthin-producing *Celosia cristata*, a close relative of amaranth [39]. Moreover, a corresponding UDP-glucuronic acid: cDOPA 5-glucoside glucuronosyltransferase activity needed for amaranthine's final biosynthetic step has been detected, further supporting the notion that modification with the glucuronic acid moiety occurs at glucosylated cDOPA prior to the condensation step with betanidin [18], [40]. DODA, cDOPA synthase and tyrosinase are considered crucial enzymes for betacyanin synthesis [18], although their importance appears to vary in a species-dependent manner [41]–[43].

Betalains are believed to play important roles in plant physiology and to modulate optical attraction for pollinators and seed dispersers. Their accumulation has been demonstrated to be diversely regulated by many endogenous and exogenous factors [14], [44], although some workers have proposed that amaranthine is an intermediate involved in conversion of cellular nitrogen compounds in amaranth plants [20]. More importantly, a protective reactive oxygen species (ROS) scavenging role, activated under stressful conditions, has been inferred from a number of studies [45]–[47]. Betacyanins have also been proposed to have photo-protective properties, as reported in *Mesembryanthemum crystallinum* and in *A. tricolor* [48]–[50].

In the present study, we describe the isolation of cDNA sequences of key genes of the betacyanin biosynthetic pathway in *A. hypochondriacus*. These include two *DODA* genes (*AhDODA-1* and *AhDODA-2*), a UDP–glucose: cyclo-DOPA 5-O glucosyltransferase (*AhcDOPA5-GT*) and a UDP–glucose: betanidin 5-O-glucosyltransferase (*AhB5-GT*), which suggest the possibility that both glucosylation pathways leading to betanins, believed to operate independently of each other, may be used in *A. hypochondriacus* to synthesize amaranthine. In addition, a comprehensive gene expression analysis, coupled with the DOPA oxidase tyrosinase assays, showed that these genes were induced differentially in a tissue- and genotype-specific manner in response to different stimuli. This study was meant to provide additional elements to further the understanding of the biological function and regulatory mechanisms of betacyanin biosynthesis in amaranth plants. However, part of the data generated might only be explained by considering possible alternative scenarios for the function of betacyanin-biosynthetic enzymes in these plants.

## Results

### Cloning of *A. hypochondriacus* betacyanin-biosynthetic genes

One partial and four complete cDNA sequences of enzymes involved in what are considered to be the basic steps of the betacyanin pigment biosynthetic pathway were obtained and further characterized. The phylogenetic analysis of the amino-acid sequences deduced from their cDNA sequences is shown in files S1 (AhDODA1 and AhDODA2), S2 (AhcDOPA5-GT), and S3 (AhB5-GT). The partial amino-acid sequence of AhCYP76, derived from sequence information (isotig 09513) obtained directly from the transcriptomic analysis of *Ah* mentioned above was also highly homologous to the orthologs reported in *A. cruentus* and other related species (results not shown).

### Betacyanin content variation in tissues of pigment-contrasting *A. hypochondriacus* genotypes and its relationship with DOPA oxidation tyrosinase (DOT) activity and betacyanin-biosynthetic gene expression

**Figure 1:**
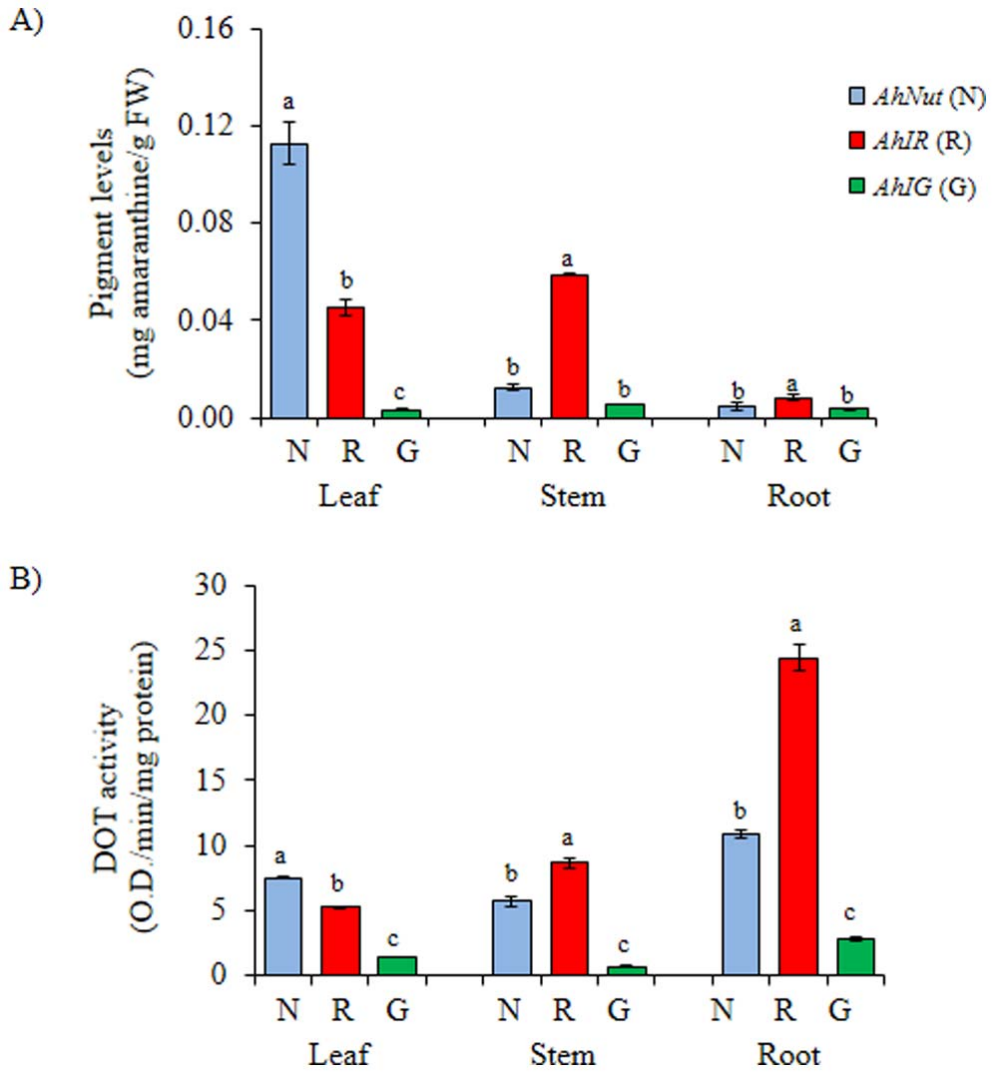
Pigment levels and tyrosinase activity in *A. hypochondriacus* plants with contrasting pigmentation patterns. Basal amaranthine (**A**) and DOPA oxidase tyrosinase activity (DOT) (**B**) levels measured in leaves, stems and roots of plants of *A. hypochondriacus* genotypes having different patterns of pigmentation: *AhNut* (predominantly betacyanic leaves, [N]; blue bars); *AhIR* (green leaves with pigmented vasculature and betacyanic stems, [R]; red bars) and *AhIG* (all tissues acyanic, [G]; green bars). Mean values ± SE are presented (n = 6). Different letters over the bars represent statistically different values at *P*≤0.05 (Tukey Kramer test). FW  =  fresh weight.

The three *A. hypochondriacus* genotypes employed in this study were chosen on the basis of their well-defined pigmentation patterns. *AhNut* presented leaves with mixed pigmented and green sectors. Its stems and roots were weakly pigmented. *AhIR* had strongly pigmented stems and moderately pigmented roots, with green leaves having pigmented vasculature, whereas *AhIG* was completely acyanic, with no visible evidence of red/purple pigmentation. The pigmentation patterns coincided with the quantitative analysis of betacyanin pigments shown in Figure 1A.

DOPA oxidation tyrosinase (DOT) activity did not always coincide with the basal betacyanin content present in different tissues (Figure 1B). A positive association between betacyanin levels and DOT activity was found in leaves of *AhNut* and in the highly pigmented stems of *AhIR*. This was also observed in all green tissues of *AhIG*, in which low DOT activity levels were detected. On the other hand, an inverse relationship between pigmentation and DOT activity was observed in roots of *AhNut* and *AhIR*, in which the low betacyanin levels contrasted with their relatively high DOT activity levels.

**Figure 2:**
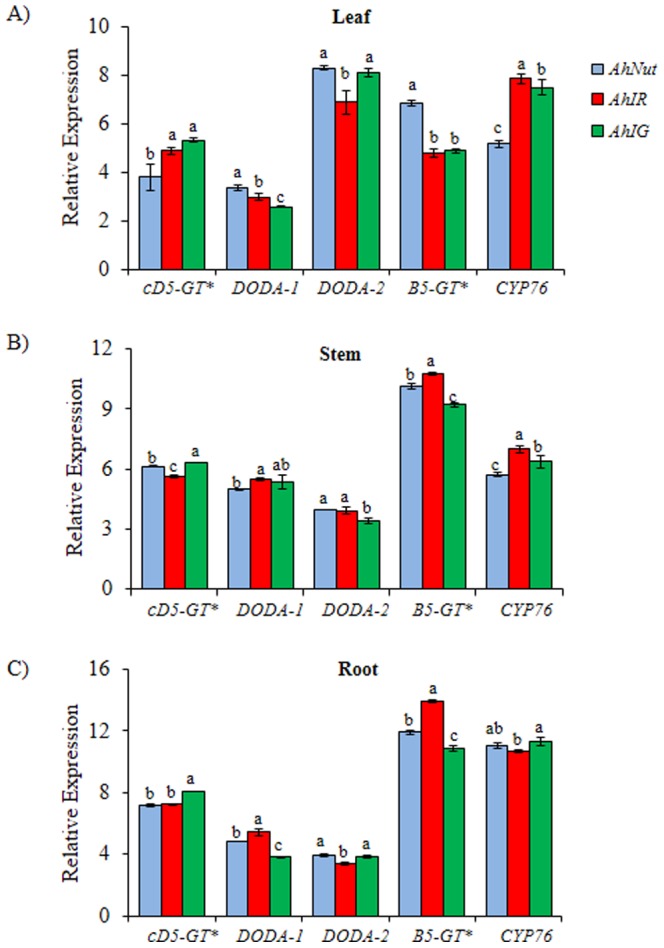
Expression levels of betacyanin biosynthetic genes in *A. hypochondriacus* plants with contrasting pigmentation patterns. Real-time quantitative polymerase chain reaction analysis of the basal expression of 5 betacyanin biosynthetic genes in **(A)** leaves, **(B)** stems and **(C)** roots of three genotypes of *A. hypochondriacus* plants differing in their pigmentation patterns, as described in Figure 1. The genes analyzed were the following: a UDP-glucose: cyclo-DOPA 5-O glucosyltransferase (*cD5-GT*), two 4, 5-DOPA-extradiol-dioxygenase genes (*DODA-1* and *DODA-2*), a UDP-glucose: betanidin 5-*O*-glucosyltransferase (*B5-GT*), and an ortholog of the red beet cytochrome P-450 *R* gene (*CYP76*). Transcript levels of these genes were normalized using *A. hypochondricus* actin and tubulin, as described in [77]. Data are means ± SE (n = 6). Different letters over the bars represent statistically different values at *P*≤0.05 (Tukey Kramer test). FW  =  fresh weight.

An analysis of the basal expression levels of betacyanin-biosynthetic genes indicated that the significantly higher expression levels of *AhDODA-1* and *AhB5-GT* correlated positively with increased betacyanin content in leaves of *AhNut*, and stems of *AhIR* (Figures 2A and B). In the latter tissues, a significantly higher expression of the *AhCYP76* gene also coincided with augmented betacyanin content (Figure 2B). Curiously, the expression levels of *AhcDOPA5-GT* tended to be high in all *AhIG* tissues tested, particularly in stems and roots, where it was significantly higher than those detected in the other two genotypes (Figures 2B and C). Conversely, the low accumulation of betacyanins coincided with significantly lower *AhB5-GT* expression levels in leaves of *AhIR* and in all *AhIG* tissues examined. Significantly lower expression of *AhDODA-1* also coincided with low betacyanin contents in leaves and roots of *AhIG* (Figures 2A and C).

### Betacyanin content variation in tissues of different *A. hypochondriacus* genotypes and its relationship with DOT activity and betacyanin-biosynthetic gene expression in plants exposed drought and salt stress

**Figure 3:**
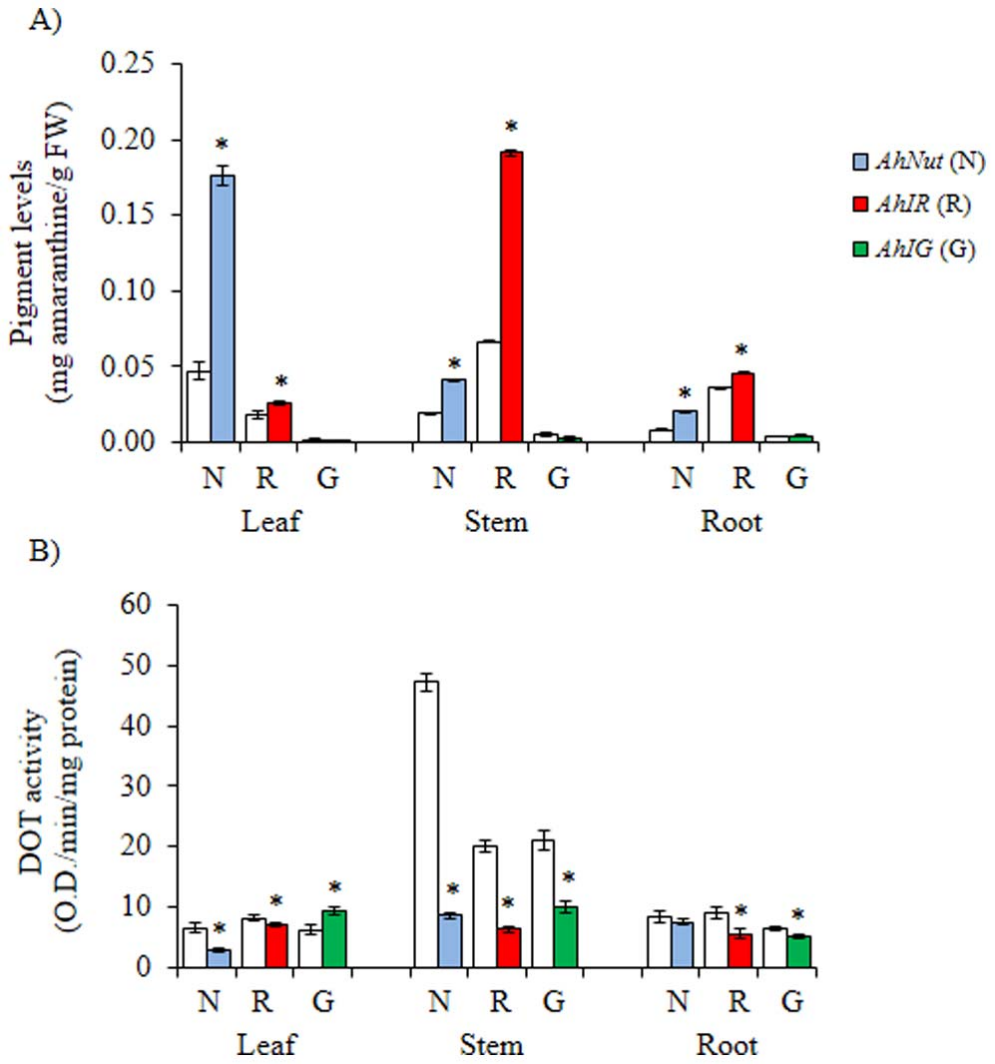
Changes in pigment levels and tyrosinase activity in water-stressed amaranth plants with contrasting pigmentation patterns. Amaranthine **(A)** and DOPA oxidase tyrosinase (DOT) activity **(B)** levels measured in leaves, stems and roots of water-stressed plants of *A. hypochondriacus* genotypes (*AhNut* [N], *AhIR* [R] and *AhIG* [G]) having different patterns of pigmentation, as described in Figure 1. Mean values ± SE in control (C; empty bars) and treated (colored bars) are presented (n = 6). Asterisks over the bars represent statistically different values at *P*≤0.05 (Tukey Kramer test). Experiments were performed twice, and representative results are shown. FW  =  fresh weight.

Betacyanin levels were differently affected in *Ah* plants subjected to drought- or salt-stress. Drought-stress induced the accumulation of betacyanins in all tissues of *AhNut* and *AhIR* plants examined. The inductive effect was predominantly strong in leaves of *AhNut* and stems of *AhIR*, in which approximately 3-fold increases in betacyanin content were detected. In contrast, pigment content in acyanic *AhIG* plants tended to decrease to even lower levels, particularly in leaves and stems (Figure 3A). In contrast to untreated plants, DOT levels significantly decreased in the majority of tissues examined, irrespective of genotype, in plants subjected to drought stress. The only exception corresponded to leaves of *AhIG*, where a slight but significant increase in DOT activity was detected. The fall in DOT activity was particularly noticeable in plant stems (Figure 3B).

The gene expression assays performed with tissues of plants subjected to drought stress, presented in Table 1, were perplexing in some measure. For instance, all five betacyanin biosynthetic genes analyzed in leaves of *AhNut* tended to be down-regulated, in particular *AhDODA-2* and *AhCYP76*, which were strongly suppressed. This occurred even though drought stress caused a ca. 3-fold betacyanin accumulation in these tissues. Also unexpected was the finding that gene expression patterns in stems of both *AhNut* and *AhIG* drought stressed plants was very similar. In them, *AhDODA-1*, *AhB5-GT* and *AhcDOPA5-GT*, were induced, in particular *AhB5-GT* (in both genotypes) and *AhDODA-1* (in *AhIG*), whereas *AhCYP76* was strongly repressed in both genotypes. The similarity of these gene expression patterns did not agree with the contrasting results shown in Figure 3A, where betacyanin levels in response to drought-stress increased in one genotype (*AhNut*), and were reduced to even lower levels in the other (*AhIG*). Conversely, the expression level of these genes in *AhIR* was more congruent with the changes in betacyanin content produced by drought-stress. Here, both glucosyltransferase genes were induced in stems and roots, whereas a tissue-specific expression of the *AhDODA* isoforms was observed, with *AhDODA-1* and *-2* being induced in roots and stems of drought stressed *AhIR* plants, respectively. Also, the induced expression of *AhCYP76* in roots of *AhIR* coincided with significantly increased betacyanin contents in response to drought stress.

**Table 1:** Expression of betacyanin biosynthetic genes in response to water stress.

Gene	Tissue	*AhNut* 1	*AhIR* 1	*AhIG* 1
*AhcDOPA5-GT*	Leaf	0.69±0.06	1.18±0.13	0.57±0.02
	Root	**1.47±0.02**	**2.08±0.04**	0.76±0.07
	Stem	**2.06±0.07**	**1.95±0.13**	**1.67±0.09**
*AhDODA-1*	Leaf	0.62±0.07	0.99±0.02	0.58±0.02
	Root	0.90±0.11	**3.63±0.81**	1.16±0.09
	Stem	**2.16±0.18**	1.21±0.04	**8.87±0.68**
*AhDODA-2*	Leaf	*0.16*±*0.03*	*0.41*±*0.04*	1.11±0.06
	Root	0.72±0.02	0.73±0.06	0.76±0.05
	Stem	0.78±0.02	**2.02±0.05**	1.29±0.04
*AhB5-GT*	Leaf	0.67±0.03	0.97±0.08	0.59±0.05
	Root	**2.94±0.48**	**16.75±0.65**	0.78±0.02
	Stem	**11.66±0.75**	**3.14±0.21**	**5.28±0.12**
*AhCYP76*	Leaf	*0.07*±*0.00*	0.54±0.04	**2.48±0.31**
	Root	0.61±0.01	**2.16±0.18**	*0.10*±*0.00*
	Stem	*0.12*±*0.01*	0.70±0.05	*0.08*±*0.00*

Relative expression levels^2^ were determined in leaves, stems and roots of *A. hypochondriacus* plants, with contrasting pigmentation patterns, subjected to water stress. Induced or repressed levels of expression (i.e. relative expression ≥1.5 or ≤0.5) are shown in bold text and italics, respectively.

1 The genotypes examined in this study were *Ah* cv. Nutrisol (*AhNut*; with predominantly betacyanic leaves), *Ah* India Red (*AhIR*; with predominantly betacyanic stems) and *Ah* India Green (*AhIG*; with all tissues acyanic).

2 The fold change in the expression of the target genes was calculated using the 2^−ΔΔCt^ method according to [76].

**Figure 4:**
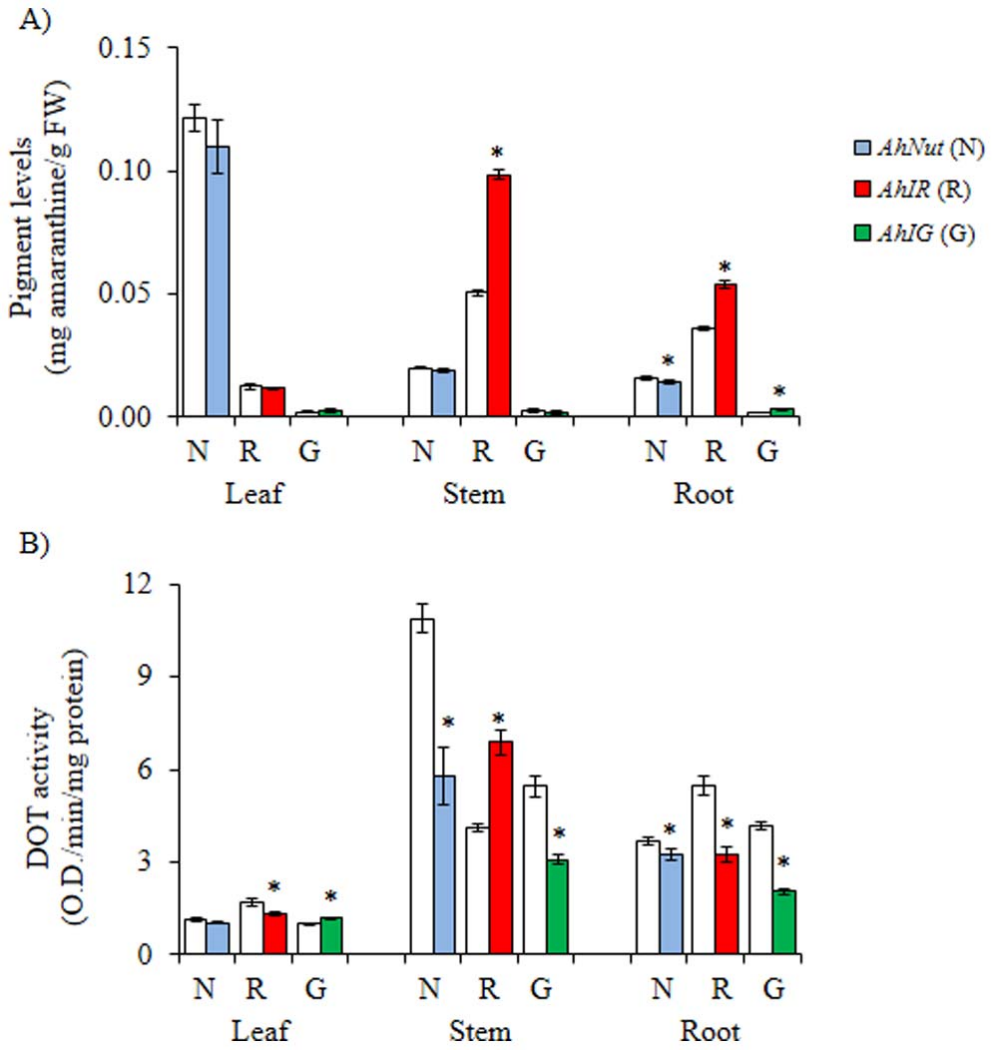
Changes in pigment levels and tyrosinase activity in salt-stressed amaranth plants with contrasting pigmentation patterns. Amaranthine **(A)** and DOPA oxidase tyrosinase (DOT) activity **(B)** levels measured in leaves, stems and roots of salt-stressed plants of *A. hypochondriacus* genotypes (*AhNut* [N], *AhIR* [R] and *AhIG* [G]) having different patterns of pigmentation, as described in Figure 1. Mean values ± SE in control (C; empty bars) and treated (colored bars) are presented (n = 6). Asterisks over the bars represent statistically different values at *P*≤0.05 (Tukey Kramer test). Experiments were performed twice, and representative results are shown. FW  =  fresh weight.

*AhNut* and *AhIR* plants responded differently to salt-stress. *AhNut* showed a tendency to accumulate less betacyanin pigments in all tissues tested in response to this stimulus, which was significant in roots, whereas betacyanin content in stems and roots of salt-stressed *AhIR* plants underwent a significant increase. In line with their acyanic phenotype, the low levels of betacyanin pigments in *AhIG* plants were not further altered by salt stress, although a very slight but significant increase was detected in roots (Figure 4A). Similar to what was observed in drought-stressed plants, DOT activity levels were reduced or remained unchanged in response to salt-stress. The only exceptions were observed in stems of *AhIR* plants and leaves of *AhIG* plants, where a significant increase in DOT activity was detected (Figure 4B).

An important difference between DOT control levels, which were similar for the three genotypes in both abiotic stress experiments (Figures 3B and 4B), with those shown in Figure 1B, in which evident differences between genotypes were detected, might have been caused by differences in light intensity and/or temperature. This possibility arises from the fact that the former experiments were performed in conditioned growth chambers, whereas the latter control plants (and also those used in the herbivory experiments [see below]) were maintained in a greenhouse under natural conditions of light and temperature.

The betacyanin-biosynthetic gene expression patterns shown in Table 2 were, once again, un-linked to betacyanin content, at least in salt-stressed *AhNut* plants. For instance, all genes examined, except for a few exceptions, were induced in response to this stressful condition in tissues of *AhNut* plants, even when betacyanin contents tended to remain constant or decrease, as shown in Figure 4A. A similar scenario was observed in salt-stressed *AhIR* plants, in which the induced accumulation of betacyanin in roots was, in general, not supported by enhanced betacyanin-biosynthetic gene expression. On the other hand, the induced expression of these genes, except *AhDODA-2*, in stems of these plants correlated with betacyanin accumulation. Similarly, the sporadic expression pattern of these genes in response to salt-stress in *AhIG* plants, most of which remained unchanged or were down-regulated (particularly *AhcDOPA5-GT* and *AhDODA-1*) was in agreement with their low and unchanging betacyanin content. A curious observation was that both abiotic stresses induced a small but significant increase in DOT activity in leaves of *AhIG* plants.

**Table 2:** Expression of betacyanin biosynthetic genes in response to salt stress.

Gene	Tissue	*AhNut* 1	*AhIR* 1	*AhIG* 1
*AhcDOPA5-GT*	Leaf	**1.73±0.08**	1.31±0.09	1.02±0.11
	Root	**2.32±0.09**	**2.02±0.17**	**1.49±0.05**
	Stem	1.14±0.07	**3.36±0.13**	*0.41*±*0.01*
*AhDODA-1*	Leaf	**1.80±0.15**	0.76±0.08	1.11±0.10
	Root	**1.47±0.04**	0.77±0.06	0.83±0.01
	Stem	**1.51±0.11**	**1.83±0.09**	*0.39*±*0.02*
*AhDODA-2*	Leaf	**2.67±0.10**	0.84±0.07	1.40±0.10
	Root	**2.01±0.01**	0.93±0.04	**3.44±0.18**
	Stem	**3.27±0.20**	0.96±0.08	0.78±0.03
*AhB5-GT*	Leaf	**2.25±0.13**	*0.38*±*0.05*	**1.92±0.07**
	Root	0.92±0.04	*0.14*±*0.01*	0.57±0.06
	Stem	**1.89±0.31**	**4.57±0.23**	0.71±0.01
*AhCYP76*	Leaf	0.80±0.04	0.97±0.06	*0.50*±*0.05*
	Root	**1.72±0.08**	0.86±0.06	**1.57±0.11**
	Stem	**2.11±0.16**	**2.28±0.24**	1.04±0.06

Relative expression levels^2^ were determined in leaves, stems and roots of *A. hypochondriacus* plants, with contrasting pigmentation patterns, subjected to salt stress. Induced or repressed levels of expression (i.e. relative expression ≥1.5 or ≤0.5) are shown in bold text and italics, respectively.

1 The genotypes examined in this study were *Ah* cv. Nutrisol (*AhNut*; with predominantly betacyanic leaves), *Ah* India Red (*AhIR*; with predominantly betacyanic stems) and *Ah* India Green (*AhIG*; with all tissues acyanic).

2 The fold change in the expression of the target genes was calculated using the 2^−ΔΔCt^ method according to [76].

### Betacyanin content variation in tissues of pigment-contrasting *A. hypochondriacus* genotypes and its relationship with DOT activity and betacyanin-biosynthetic gene expression in plants exposed to insect herbivory

**Figure 5:**
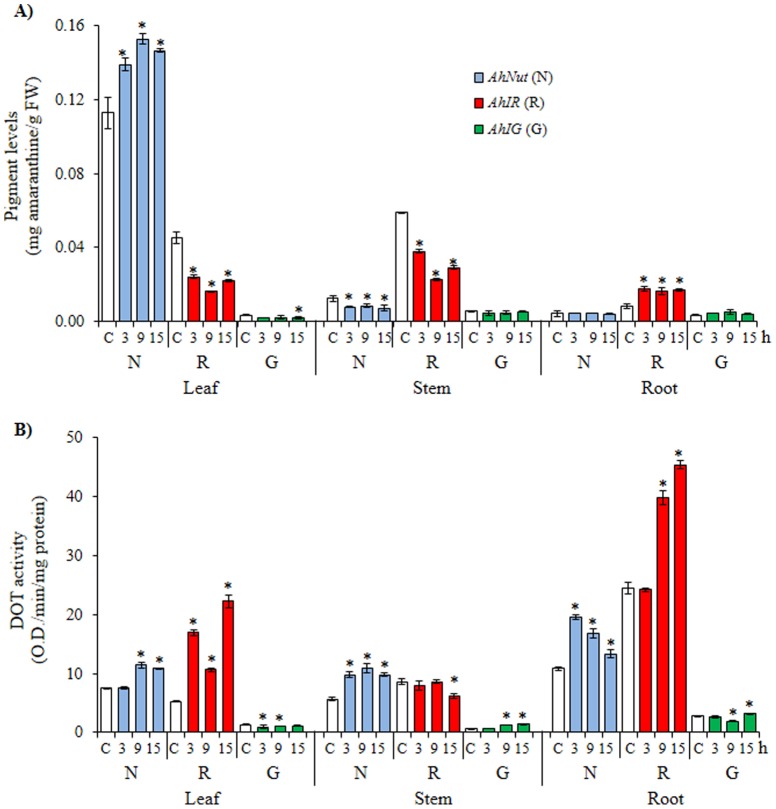
Changes in pigment levels and tyrosinase activity in amaranth plants subjected to *discontinuous* insect herbivory. Amaranthine (**A**) and DOPA oxidase tyrosinase (DOT) activity (**B**) levels measured in leaves, stems and roots of plants of *A. hypochondriacus* genotypes (*AhNut* [N], *AhIR* [R] and *AhIG* [G]) having different patterns of pigmentation, as described in Figure 1, and subjected to *discontinuous* (samples were taken on removing the larvae) insect herbivory for 3, 9 and 15 h. Mean values ± SE in control (C: empty bars) and treated (colored bars) plants are presented (n = 6). Asterisks over the bars represent statistically different values at *P*≤0.05 (Dunnetts test). Experiments were performed twice, and representative results are shown. FW  =  fresh weight.

Two variations of the insect herbivory experiment were performed. In one, plants were exposed to *Spoladea recurvalis* larvae for different lapses of time (3 to 18 h) and were then removed (“*discontinuous herbivory*”, or DH). Sampling of the plant tissues was done concomitant with larval removal. The effect on betacyanin content by this modality of insect herbivory was variable and was also tissue and genotype-specific. DH induced the accumulation of betacyanins in leaves of *AhNut* and roots of *AhIR*. In all other tissues and genotypes examined DH either had no effect on betacyanin content or caused a reduction in pigment levels. Reductions were recorded in leaves and stems of *AhIR* and in stems of *AhNut* (Figure 5A). DOT activity was generally induced by DH in all betacyanic tissues examined and coincided with the accumulation of pigment in leaves and roots of *AhNut* and *AhIR*, respectively (Figure 5B).

The gene expression analyses shown in Table 3 indicate that DH caused a strong induction of both glucosyltransferases and of *AhDODA-2* in the leaves of all three genotypes tested but had a neutral to negative effect on the other two genes examined. The repressive effect was particularly strong on the *AhCYP76* gene. The expression pattern recorded in stems only resembled that in leaves in the mostly neutral effect on the *AhDODA-1* gene and in the strong down-regulation of the *AhCYP46* gene (Table 4). The three other genes showed a sporadic increase in expression in stems, mostly observed at relatively late time points. In contrast, root gene expression resembled that recorded in leaves (Table 5), except for the sporadic up-regulation of the *Ah-DODA-1* and *AhCYP76* genes in of both *AhNut* and *AhIR*, and the early and temporal induction of *AhB5-GT* in *AhNut* and *AhIG*, which contrasted with its strong repression in *AhIR*.

**Table 3:** Expression of betacyanin biosynthetic genes in response to *discontinuous* insect herbivory.

Gene	h^1^	*AhNut* 2	*AhIR* 2	*AhIG* 2
*AhcDOPA5-GT*	3	1.34±0.08	**3.15±0.07**	**1.79±0.13**
	6	0.98±0.06	1.12±0.02	**1.86±0.04**
	9	**1.56±0.05**	**1.96±0.13**	**2.14±0.17**
	12	**2.26±0.10**	**5.00±0.22**	**5.32±0.12**
	15	**4.47±0.34**	**3.14±0.07**	**5.50±0.76**
	18	**2.05±0.14**	**1.74±0.15**	**2.16±0.17**
*AhDODA-1*	3	0.97±0.04	1.44±0.06	0.62±0.03
	6	0.66±0.04	0.96±0.04	*0.50*±*0.05*
	9	0.54±0.03	1.00±0.06	0.74±0.02
	12	0.61±0.00	**2.12±0.02**	0.90±0.03
	15	*0.40*±*0.03*	1.20±0.04	*0.27*±*0.01*
	18	*0.27*±*0.03*	0.86±0.05	*0.18*±*0.02*
*AhDODA-2*	3	1.22±0.03	**5.59±0.45**	**1.67±0.01**
	6	**1.50±0.02**	**3.63±0.05**	**3.97±0.20**
	9	**1.73±0.02**	**2.28±0.34**	**2.04±0.12**
	12	**2.24±0.07**	**7.48±0.32**	**3.48±0.31**
	15	**4.25±0.07**	**8.29±0.74**	**8.03±0.87**
	18	**2.53±0.07**	**4.28±0.19**	**2.62±0.16**
*AhB5-GT*	3	**1.96±0.03**	**8.75±1.00**	**1.86±0.06**
	6	**3.07±0.48**	**2.85±0.23**	**2.52±0.39**
	9	**2.31±0.75**	**4.18±0.20**	**1.83±0.13**
	12	**7.22±0.07**	**24.46±0.61**	**17.05±0.99**
	15	**34.64±0.07**	**63.51±5.77**	**17.14±0.62**
	18	**16.65±0.07**	**30.96±2.78**	**4.05±0.38**
*AhCYP76*	3	*0.19*±*0.00*	*0.46*±*0.08*	*0.32*±*0.03*
	6	*0.02*±*0.00*	1.14±0.07	0.96±0.06
	9	*0.12*±*0.01*	*0.06*±*0.01*	0.83±0.03
	12	*0.14*±*0.01*	*0.10*±*0.02*	0.73±0.03
	15	*0.02*±*0.00*	*0.34*±*0.01*	*0.04*±*0.01*
	18	*0.06*±*0.01*	*0.01*±*0.00*	*0.00*±*0.00*

Relative expression levels^3^ were determined in leaves of *A. hypochondriacus* plants, with contrasting pigmentation patterns, subjected to insect herbivory. Induced or repressed levels of expression (i.e. relative expression ≥1.5 or ≤0.5) are shown in bold text and italics, respectively.

1 h =  time, in hours, spent on the plant by the feeding larvae before they were removed and the tissues sampled.

2 The genotypes examined in this study were *Ah* cv. Nutrisol (*AhNut*; with predominantly betacyanic leaves), *Ah* India Red (*AhIR*; with predominantly betacyanic stems) and *Ah* India Green (*AhIG*; with all tissues acyanic).

3 The fold change in the expression of the target genes was calculated using the 2^−ΔΔCt^ method according to [76].

**Table 4:** Expression of betacyanin biosynthetic genes in response to *discontinuous* insect herbivory.

Gene	h^1^	*AhNut* 2	*AhIR* 2	*AhIG* 2
*AhcDOPA5-GT*	3	0.75±0.06	0.66±0.05	1.06±0.06
	6	0.80±0.01	0.86±0.01	1.16±0.02
	9	**1.47±0.01**	1.12±0.17	**1.70±0.00**
	12	**1.79±0.15**	1.34±0.08	0.83±0.01
	15	**1.38±0.04**	**2.10±0.15**	0.88±0.01
	18	**1.89±0.06**	**2.11±0.18**	**2.43±0.28**
*AhDODA-1*	3	1.33±0.17	*0.39*±*0.01*	1.41±0.11
	6	0.65±0.04	0.85±0.07	0.54±0.02
	9	0.80±0.02	0.82±0.01	0.64±0.01
	12	0.62±0.03	0.66±0.03	*0.25*±*0.01*
	15	1.12±0.04	0.77±0.04	0.51±0.01
	18	0.51±0.02	0.53±0.03	*0.34*±*0.04*
*AhDODA-2*	3	1.35±0.06	0.60±0.04	**2.24±0.16**
	6	0.89±0.07	*0.47*±*0.04*	1.02±0.02
	9	**1.84±0.11**	0.51±0.04	0.85±0.09
	12	0.80±0.02	0.71±0.01	0.69±0.02
	15	**2.73±0.40**	1.13±0.02	0.67±0.04
	18	0.59±0.04	0.72±0.04	1.20±0.03
*AhB5-GT*	3	1.00±0.04	0.78±0.03	**2.43±0.06**
	6	0.92±0.10	1.19±0.09	**2.57±0.17**
	9	0.60±0.03	1.18±0.05	**2.24±0.27**
	12	**2.05±0.18**	1.29±0.17	1.05± 0.09
	15	1.38±0.07	**4.32±0.35**	1.33± 0.20
	18	0.70±0.01	**2.21±0.14**	**2.72±0.16**
*AhCYP76*	3	0.97±0.05	*0.47*±*0.01*	0.76±0.03
	6	*0.10*±*0.01*	*0.08*±*0.00*	*0.33*±*0.01*
	9	*0.09*±*0.00*	*0.03*±*0.01*	*0.14*±*0.01*
	12	*0.23*±*0.01*	0.55±0.04	*0.04*±*0.01*
	15	*0.02*±*0.00*	*0.08*±*0.01*	*0.02*±*0.00*
	18	*0.02*±*0.00*	*0.02*±*0.00*	*0.06*±*0.00*

Relative expression levels^3^ were determined in stems of *A. hypochondriacus* plants, with contrasting pigmentation patterns, subjected to insect herbivory. Induced or repressed levels of expression (i.e. relative expression ≥1.5 or ≤0.5) are shown in bold text and italics, respectively.

1 h =  time, in hours, spent on the plant by the feeding larvae before they were removed and the tissues sampled.

2 The genotypes examined in this study were *Ah* cv. Nutrisol (*AhNut*; with predominantly betacyanic leaves), *Ah* India Red (*AhIR*; with predominantly betacyanic stems) and *Ah* India Green (*AhIG*; with all tissues acyanic).

3 The fold change in the expression of the target genes was calculated using the 2^−ΔΔCt^ method according to [76].

**Table 5:** Expression of betacyanin biosynthetic genes in response to *discontinuous* insect herbivory.

Gene	h^1^	*AhNut* 2	*AhIR* 2	*AhIG* 2
*AhcDOPA5-GT*	3	**3.15±0.10**	**2.42±0.20**	1.23±0.07
	6	**5.63±0.34**	**3.96±0.13**	**3.50±0.21**
	9	**7.30±0.33**	**16.46±0.35**	**8.44±0.41**
	12	**1.48±0.13**	**13.83±0.81**	**2.82±0.18**
	15	**7.56±0.54**	**2.44±0.05**	**12.52±0.11**
	18	**3.41±0.20**	**4.82±0.35**	**10.63±1.05**
*AhDODA-1*	3	1.18±0.01	0.62±0.04	*0.44*±*0.02*
	6	1.46±0.03	1.00±0.08	*0.30*±*0.01*
	9	**1.53±0.09**	**2.19±0.09**	*0.50*±*0.09*
	12	0.67±0.02	**1.89±0.07**	*0.39*±*0.01*
	15	1.37±0.11	**1.92±0.08**	0.66±0.08
	18	0.96±0.08	1.42±0.06	0.57±0.03
*AhDODA-2*	3	**1.81±0.10**	*0.47*±*0.03*	**2.11±0.11**
	6	**6.98±0.51**	**2.92±0.07**	**4.24±0.30**
	9	**4.91±0.04**	**3.75±0.11**	**4.73±0.00**
	12	0.72±0.06	0.81±0.01	1.18±0.04
	15	**4.84±0.06**	**1.77±0.00**	**8.79±1.00**
	18	**1.55±0.06**	**2.21±0.12**	**3.07±0.22**
*AhB5-GT*	3	**1.77±0.14**	*0.17*±*0.05*	**3.10±0.20**
	6	**4.42±0.22**	*0.34*±*0.01*	**3.15±0.20**
	9	**6.49±0.50**	*0.17*±*0.01*	**3.83±0.10**
	12	1.19±0.08	*0.23*±*0.00*	**2.01± 0.24**
	15	1.32±0.04	1.01±0.15	0.63± 0.05
	18	0.66±0.12	*0.08*±*0.00*	1.34±0.07
*AhCYP76*	3	**3.49±0.27**	**2.20±0.05**	1.38±0.12
	6	**7.57±0.44**	1.27±0.02	1.17±0.09
	9	**1.49±0.16**	1.32±0.10	0.69±0.06
	12	1.20±0.07	0.72±0.01	*0.35*±*0.02*
	15	0.54±0.05	**1.93±0.16**	*0.22*±*0.01*
	18	*0.20*±*0.01*	*0.05*±*0.00*	*0.05*±*0.00*

Relative expression levels^3^ were determined in roots of *A. hypochondriacus* plants, with contrasting pigmentation patterns, subjected to insect herbivory. Induced or repressed levels of expression (i.e. relative expression ≥1.5 or ≤0.5) are shown in bold text and italics, respectively.

1 h =  time, in hours, spent on the plant by the feeding larvae before they were removed and the tissues sampled.

2 The genotypes examined in this study were *Ah* cv. Nutrisol (*AhNut*; with predominantly betacyanic leaves), *Ah* India Red (*AhIR*; with predominantly betacyanic stems) and *Ah* India Green (*AhIG*; with all tissues acyanic).

3 The fold change in the expression of the target genes was calculated using the 2^−ΔΔCt^ method according to [76].

**Figure 6:**
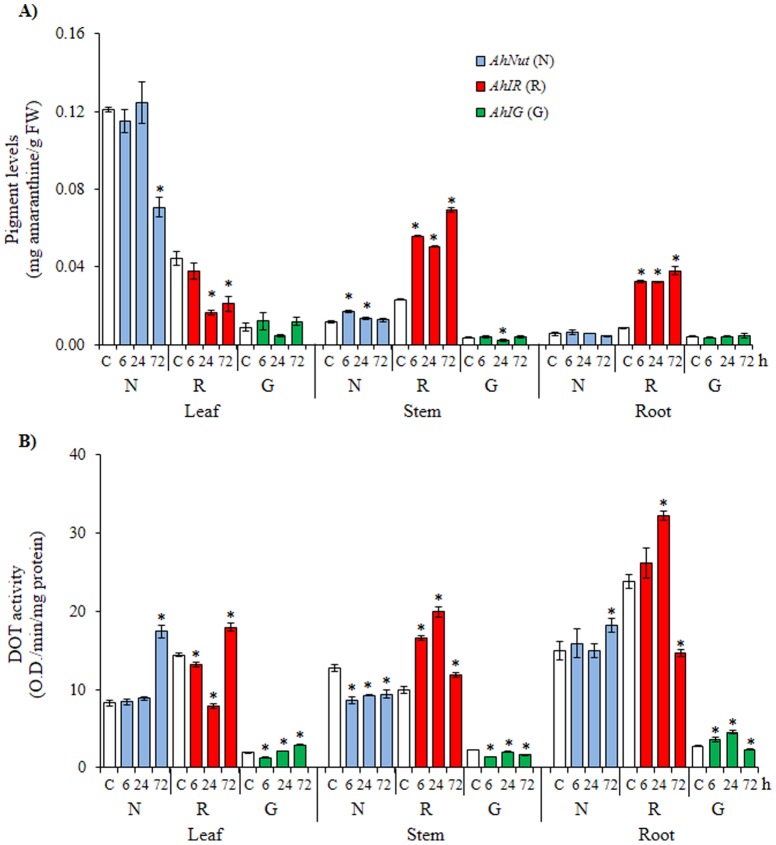
Changes in pigment levels and tyrosinase activity in amaranth plants subjected to *continuous* insect herbivory. Amaranthine (**A**) and DOPA oxidase tyrosinase (DOT) activity (**B**) levels measured in leaves, stems and roots of plants of *A. hypochondriacus* genotypes (*AhNut* [N], *AhIR* [R] and *AhIG* [G]) having different patterns of pigmentation, as described in Figure 1, and subjected to *continuous* insect herbivory for 18 h and then sampled at 6, 24 and 72 h after larval removal. Mean values ± SE in control (C: empty bars) and treated (T: colored bars) plants are presented (n = 6). Asterisks over the bars represent statistically different values at *P*≤0.05 (Dunnetts test). Experiments were performed twice, and representative results are shown. FW  =  fresh weight.

In the second variation of the insect herbivory experiment (“*continuous herbivory*”, or CH), larvae were allowed to feed on the leaves of the plants for 18 h and were then removed. Sampling of the plant tissues was done at different time points (1 to 72 h) after larval removal. In most cases, betacyanin content in leaves gradually declined or remained unchanged after larval removal in the three genotypes. The exceptions were the significant accumulation of pigment in stems of *AhNut* and *AhIR* and in roots of *AhIR* (Figure 6A). DOT activity was induced at late time points in leaves (i.e. 72 h), which did not coincide with augmented pigmentation. It had an inverse correlation with pigment accumulation in stems of *AhNut*, but not in those of *AhIR*, where high DOT activity was consistent with increased pigmentation. Also, DOT induction, observed 24 h after larval removal, coincided with pigment accumulation in roots of *AhIR*. On the other hand, the down-regulation of DOT activity detected 72 h after larval removal in roots of *AhNut* and *AhIR* did not coincide with the augmented pigmentation detected (Figure 6B). Curiously, CH had an inductive effect on DOT activity in leaves and roots of *AhIG*.

Gene expression in response to CH varied from that observed in DH, as shown in Tables 6 to 8. The expression levels of all genes examined tended to decline in leaves in the absence of direct insect stimulus. The effect was more noticeable in *AhNut* for *AhcDOPA5-GT* and *AhCYP76*. A similar decline in the expression of these genes was also detected in the other two genotypes, except for the irregular up-regulation of *AhcDOPA5-GT* and the induction of *AhCYP76*, particularly in *AhIR* (Table 6). Gene expression changed moderately in stems and drastically in roots, where it became stronger and more regular in all genotypes tested. The only exceptions were observed in *AhIG*, in which a neutral to negative effect was produced in the expression of both glucosyltransferase genes in stems, and of *AhDODA-1* in roots (Tables 7 and 8).

**Table 6:** Expression of betacyanin biosynthetic genes in response to *continuous* insect herbivory.

Gene	h^1^	*AhNut* 2	*AhIR* 2	*AhIG* 2
*AhcDOPA5-GT*	1	0.54±0.04	**1.64±0.12**	**1.59±0.07**
	6	*0.28*±*0.08*	1.39±0.07	0.54±0.03
	12	*0.43*±*0.01*	1.19±0.08	0.70±0.03
	24	0.61±0.02	**2.14±0.01**	1.00±0.06
	48	*0.37*±*0.04*	**1.86±0.13**	**1.68±0.01**
	72	*0.25*±*0.00*	0.79±0.00	0.71±0.03
*AhDODA-1*	1	0.57±0.01	1.00±0.09	0.51±0.01
	6	**2.67±0.09**	1.15±0.06	0.76±0.07
	12	**3.55±0.14**	**2.40±0.24**	**1.61±0.03**
	24	1.19±0.02	1.29±0.09	0.56±0.03
	48	0.60±0.04	0.72±0.05	1.12±0.03
	72	0.63±0.05	*0.34*±*0.02*	0.53±0.04
*AhDODA-2*	1	**1.63±0.17**	**1.54±0.09**	**1.75±0.02**
	6	0.59±0.06	*0.35*±*0.02*	*0.24*±*0.01*
	12	0.89±0.03	0.98±0.06	0.45±0.03
	24	*0.44*±*0.02*	1.00±0.05	**1.58±0.04**
	48	*0.49*±*0.03*	0.74±0.05	0.99±0.06
	72	0.61±0.02	*0.34*±*0.02*	*0.40*±*0.03*
*AhB5-GT*	1	**2.12±0.30**	**1.51±0.13**	0.86±0.01
	6	1.25±0.13	*0.28*±*0.02*	*0.20*±*0.01*
	12	0.55±0.02	*0.23*±*0.00*	*0.29*±*0.02*
	24	**6.37±0.38**	0.95±0.06	1.13±0.06
	48	1.43±0.05	*0.40*±*0.03*	*0.42*±0.01
	72	1.18±0.08	0.62±0.02	0.58±0.02
*AhCYP76*	1	*0.50*±*0.01*	0.58±0.12	*0.07*±*0.00*
	6	*0.14*±*0.01*	**28.03±1.29**	**5.79±0.48**
	12	1.27±0.08	**24.16±1.12**	1.24±0.15
	24	*0.18*±*0.00*	0.51±0.04	*0.08*±*0.03*
	48	*0.19*±*0.01*	**1.56±0.06**	4.56±0.26
	72	*0.12*±*0.01*	**12.67±0.70**	*0.19*±*0.03*

Relative expression levels^3^ were determined in leaves of *A. hypochondriacus* plants, with contrasting pigmentation patterns, subjected to insect herbivory. Induced or repressed levels of expression (i.e. relative expression ≥1.5 or ≤0.5) are shown in bold text and italics, respectively.

1 h =  time, in hours, when the tissues were sampled after an 18 h period of continuous insect feeding.

2 The genotypes examined in this study were *Ah* cv. Nutrisol (*AhNut*; with predominantly betacyanic leaves), *Ah* India Red (*AhIR*; with predominantly betacyanic stems) and *Ah* India Green (*AhIG*; with all tissues acyanic).

3 The fold change in the expression of the target genes was calculated using the 2^−ΔΔCt^ method according to [76].

**Table 7:** Expression of betacyanin biosynthetic genes in response to *continuous* insect herbivory.

Gene	h^1^	*AhNut* 2	*AhIR* 2	*AhIG* 2
*AhcDOPA5-GT*	1	1.34±0.07	*0.37*±*0.02*	1.10±0.04
	6	1.33±0.06	1.10±0.15	*0.39*±*0.01*
	12	*0.48*±*0.04*	0.56±0.03	*0.47*±*0.04*
	24	**1.70±0.17**	0.90±0.04	1.07±0.02
	48	0.85±0.05	**2.13±0.37**	0.81±0.03
	72	*0.47*±*0.01*	*0.49*±*0.04*	0.68±0.06
*AhDODA-1*	1	1.25±0.02	**1.96±0.07**	**3.53±0.42**
	6	**2.54±0.22**	**2.01±0.20**	**1.62±0.03**
	12	**2.20±0.18**	**2.37±0.11**	**2.10±0.08**
	24	0.97±0.12	0.84±0.04	**1.63±0.14**
	48	1.21±0.03	**1.73±0.10**	**2.07±0.14**
	72	1.00±0.03	1.17±0.07	1.39±0.07
*AhDODA-2*	1	0.62±0.01	1.32±0.05	1.00±0.08
	6	**2.07±0.04**	0.73±0.02	0.88±0.04
	12	0.65±0.05	0.81±0.08	0.71±0.07
	24	0.60±0.03	1.09±0.13	**1.87±0.07**
	48	0.98±0.03	**2.90±0.27**	0.73±0.06
	72	1.06±0.06	**1.73±0.06**	*0.33*±*0.02*
*AhB5-GT*	1	**1.78±0.11**	**3.02±0.49**	1.08±0.01
	6	**2.91±0.31**	**2.08±0.15**	*0.46*±*0.02*
	12	0.53±0.04	**1.95±0.16**	*0.40*±*0.02*
	24	**2.85±0.19**	1.17±0.11	1.08± 0.08
	48	*0.44*±*0.03*	1.25±0.04	*0.42*± *0.01*
	72	0.88±0.04	**3.48±0.10**	0.57±0.08
*AhCYP76*	1	*0.07*±*0.00*	*0.35*±*0.01*	0.79±0.07
	6	**3.25±0.25**	**29.07±1.87**	**6.76±0.35**
	12	**5.17±0.33**	**41.74±1.81**	**3.29±0.43**
	24	*0.01*±*0.00*	**11.94±0.43**	*0.05*±*0.00*
	48	*0.36*±*0.04*	**9.09±0.47**	1.24±0.02
	72	0.93±0.06	**14.07±0.93**	**1.57±0.11**

Relative expression levels^3^ were determined in stems of *A. hypochondriacus* plants, with contrasting pigmentation patterns, subjected to insect herbivory. Induced or repressed levels of expression (i.e. relative expression ≥1.5 or ≤0.5) are shown in bold text and italics, respectively.

1 h =  time, in hours, when the tissues were sampled after an 18 h period of continuous insect feeding.

2 The genotypes examined in this study were *Ah* cv. Nutrisol (*AhNut*; with predominantly betacyanic leaves), *Ah* India Red (*AhIR*; with predominantly betacyanic stems) and *Ah* India Green (*AhIG*; with all tissues acyanic).

3 The fold change in the expression of the target genes was calculated using the 2^−ΔΔCt^ method according to [76].

**Table 8:** Expression of betacyanin biosynthetic genes in response to *continuous* insect herbivory.

Gene	h^1^	*AhNut* 2	*AhIR* 2	*AhIG* 2
*AhcDOPA5-GT*	1	**1.53±0.18**	*0.36*±*0.03*	**1.56±0.10**
	6	1.11±0.04	0.87±0.04	1.02±0.01
	12	**1.62±0.07**	**3.21±0.15**	**7.26±0.27**
	24	**3.90±0.28**	**3.66±0.23**	1.41±0.03
	48	**3.40±0.21**	**4.62±0.16**	**1.78±0.17**
	72	**1.50±0.08**	**2.33±0.06**	**2.56±0.12**
*AhDODA-1*	1	**1.84±0.10**	**1.68±0.04**	0.92±0.19
	6	**1.81±0.24**	1.12±0.09	0.64±0.03
	12	**5.47±0.39**	**4.95±0.12**	0.94±0.07
	24	**1.77±0.08**	**3.27±0.21**	0.50±0.01
	48	**2.86±0.11**	**3.21±0.03**	0.61±0.01
	72	1.16±0.01	**3.04±0.19**	0.60±0.02
*AhDODA-2*	1	**1.68±0.09**	1.11±0.01	1.47±0.05
	6	**5.39±0.28**	1.18±0.07	1.47±0.03
	12	**2.74±0.07**	**5.15±0.33**	**6.39±0.23**
	24	**2.66±0.15**	**5.42±0.44**	**1.83±0.14**
	48	**5.64±0.20**	**5.14±0.31**	**5.29±0.33**
	72	**1.83±0.19**	**1.86±0.09**	**3.85±0.04**
*AhB5-GT*	1	**1.61±0.06**	1.42±0.04	**1.69±0.06**
	6	**3.16±0.24**	**13.50±0.72**	**3.49±0.45**
	12	**2.30±0.05**	**9.56±0.17**	0.54±0.02
	24	**4.68±0.00**	**15.66±0.56**	**3.89±0.34**
	48	**7.48±0.38**	**16.89±3.16**	1.36± 0.08
	72	**8.59±0.04**	**60.81±9.06**	**2.82±0.08**
*AhCYP76*	1	1.15±0.06	1.39±0.05	**4.20±0.33**
	6	**2.34±0.19**	**52.98±3.48**	**24.22±0.57**
	12	*0.13*±*0.01*	**3.06±0.33**	1.37±0.02
	24	**3.31±0.04**	**21.17±0.90**	**23.19±1.55**
	48	1.21±0.04	**5.46±0.54**	**4.54±0.02**
	72	*0.29*±*0.02*	**76.34±2.13**	**2.89±0.04**

Relative expression levels^3^ were determined in roots of *A. hypochondriacus* plants, with contrasting pigmentation patterns, subjected to insect herbivory. Induced or repressed levels of expression (i.e. relative expression ≥1.5 or ≤0.5) are shown in bold text and italics, respectively.

1 h =  time, in hours, when the tissues were sampled after an 18 h period of continuous insect feeding.

2 The genotypes examined in this study were *Ah* cv. Nutrisol (*AhNut*; with predominantly betacyanic leaves), *Ah* India Red (*AhIR*; with predominantly betacyanic stems) and *Ah* India Green (*AhIG*; with all tissues acyanic).

3 The fold change in the expression of the target genes was calculated using the 2^−ΔΔCt^ method according to [76].

## Discussion

In this study, four complete and one partial cDNA sequences of key genes in the betacyanin biosynthetic pathway in *Ah* were obtained. In addition, a comprehensive analysis of the expression pattern of these genes under diverse (a)biotic stress conditions was performed. The gene expression assays were complemented with DOT activity assays. These results were compared with betacyanin levels in different tissues with contrasting pigmentation.

As expected, AhDODA-1 clearly grouped among other DODA proteins belonging to the Caryophyllales and was most closely related with a DODA protein of *Suaeda salsa*, a native pioneering halophyte C3 Amaranthaceae plant adapted to the high salinity region in north China [51]. In contrast, AhDODA-2 did not associate clearly with any of the two well-defined Caryophyllales clusters. Its border-line aggrupation, suggests a closer similarity to DODA-like proteins present in non-betalain accumulating land plants [41]. Thus, AhDODA-2 may represent an evolutionary link between mutually exclusive anthocyanin and betanin accumulating plants. Its ambiguous position suggests that similarly to DODA-like proteins, AhDODA-2 may be involved in aspects of plant metabolism other than pigment biosynthesis (see below).

The predicted sequence of AhcDOPA5-GT complements the rather scarce number of homologous proteins in betanin accumulating plants, which have only been reported in *Mirabilis jalapa* (Nyctaginaceae) and *C. cristata* (Amaranthaceae). All three sequences clustered together, as they all belong to members of the Caryophyllales. The closer association of the *A. hypochondriacus* and *C. cristata* proteins was to be expected considering their proximate phylogenetic relationship. A similar pattern was produced by the phylogenetic analysis of AhB5-GT, which clustered firmly within the Caryophyllales clade, showing nearest homology with a B5-GT of *B. vulgaris*, a close relative of *Amaranthus*.

Visual pigmentation in the different tissues of untreated *Ah* plants coincided in most cases with their betacyanin content. The strong pigmentation of the stems of *AhR1* might have represented a superficial localization of pigments, perhaps for protective functions, similar to what was observed in cactus stems. Here, the accumulation of betacyanins in the hypodermis and outer layers of the chlorenchyma was proposed to form a protective antioxidant screen for the underlying photosystems and cortex [52]. However, the betacyanin levels detected in the betacyanic *Ah* genotypes herewith examined, were at least 2-fold lower than those reported previously in several tissues of diverse *Amaranthus* species, including *Ah* [19]. Such difference was probably caused by the less stringent extraction method used in this work, which was not designed to extract bound forms of betacyanin. These are known to associate to proteins or pectins, being only extractable by diluted alcohol and mild acid hydrolysis, respectively [20]. In this context, it is valid to suggest that part of the inconsistencies observed in this work between pigment levels, DOT activity and gene expression could have been influenced by this experimental detail.

Except for a few cases, the basal levels of the DOT activity were generally found to coincide with the betacyanin content present in the different tissues of the *Ah* genotypes examined. However, the DOT activities detected in leaves of *AhNut* and roots of *AhIR*, although significantly higher than those found in similarly tested tissues of other genotypes, were disproportionate with the pigment levels in these tissues. The reason for such disparity remains to be determined, although it showed a similar tendency to the one observed in *Ptilotus* flowers, where no correlation between DOT activity and betalain content was found in several cultivars studied [53]. In the latter case, it was suggested that the DOT assay commonly utilized may not be specific for the enzyme involved in betalain synthesis. On the other hand, most of the results obtained were in accordance with reports describing a correlation with the activity of tyrosinases and pigmentation in dark-grown *S. salsa* seedlings, fruits of *Phytolacca americana*, callus cultures and plants of *Portulaca grandiflora* and red beet, and flowers of *Lampranthus productus* [24]–[31]. Moreover, a central role for tyrosinase in pigmentation was implied by findings showing that tyrosinase activity was the essential factor determining betalain biosynthesis in red vs. white cells of *P*. *americana*, both of which accumulated similar transcripts levels of two *DODA* genes [54], and that tyrosinase activity and *DODA* mRNA levels were consistent with alterations in betacyanin content in *S. salsa* seedlings due to changes in light quality [55].

A similar tendency was observed between pigmentation and the basal expression levels of the betacyanin-biosynthetic genes examined. Exceptions found were the inverse correlation between low pigmentation in *AhIG* tissues and the relatively high basal expression levels of most genes examined, particularly *AhcDOPA5-GT* and of the *AhCYP76*, the latter coding for a homologue of the cytochrome 450 enzyme involved in the oxidation of DOPA in the betalain pathway of *B. vulgaris* [32]. The only gene that was clearly down-regulated in all acyanic tissues of *AhIG*, and some others, such as leaves of *AhIR*, was *AhB5-GT.* It is tempting to speculate that the low expression levels of this gene in tissues having weak pigmentation could indicate that the glycosylation step catalyzed by this glucosyl transferase contributes to pigment stability, e.g. by protecting them from degradation by betacyanin-decolorizing enzymes similar to those previously isolated in *P. americana* [56] or by oxidation by peroxidases [15], [57]. The implications of this possibility remain to be determined, and could prove to be an important factor defining betacyanin pigment stability and/or accumulation in amaranth and other betacyanin accumulating plants. The concept is supported the emerging importance of the glycosylation process in plants, known to be an essential component of many regulatory mechanisms including modulation of biological activity of bioactive natural products and hormones and storage into specific cellular compartments [58], [59]. This possibility must be weighed, however, against other factors that are known to influence betacyanin stability and accumulation, such as developmental cues, hormones, nutrition, light, temperature, pH, metal ions, oxygen, ascorbic acid and salinity stress (see below), among others [14], [44]. Betacyanin stability is also believed to depend on Ca^2+^, Ca^2+^-regulated ion channels, and calmodulin, as suggested by dark-induced betacyanin accumulation in *S. salsa* [60]. This remains to be determined in amaranth.

Betacyanin accumulation in *Ah* varied in response to the stress applied. It was also tissue- and genotype-specific. These factors also influenced the correlation between DOT activity, gene expression and augmented pigmentation. Hence, salt stress led to pigment accumulation exclusively in *AhIR* and only in stems and roots. This result agreed with the accumulation of betacyanins in response to high salinity detected in the C3 halophyte *S. salsa*, which has been associated with protection against oxidative damage produced by this and other types of stress as well as by direct exposure of roots to H_2_O_2_ [47], [51]. However, higher betacyanin content correlated with higher DOT activity and augmented gene expression activity in stems of *AhIR* only, as shown in Figures 4A and B and Table 2. These results suggest that similarly to *S. salsa*, a putative oxidative stress signal leading to betacyanin production in salt stressed *AhIR* plants may have been perceived by the roots initially and then transferred to the stems, where it could have regulated the observed changes in gene expression and DOT activity. Conversely, the evident lack of correlation between betacyanin levels, gene expression and DOT activity detected in tissues of salt stressed *AhNut*, further underlined the influence of the genotype on betacyanin biosynthesis in *Ah*.

Water stress also led to the accumulation of betacyanins in *Ah* plants. The effect of this condition was stronger than salt stress, involving all tissues examined in both *AhIR* and *AhNut* genotypes. These results complement the scarce information regarding betacyanin accumulation in response to water stress, and were in agreement with data showing that drought significantly induced higher concentrations of total phenolics and betalains in beets, a response which was associated, similarly to salt stress, with a higher antioxidant activity [61], [62]. However, pigment accumulation did not coincide with DOT activity, which almost universally declined in response to water stress. Moreover, repressed, unchanged or irregular gene expression patterns in leaves and stems were also incompatible with pigment accumulation in both genotypes, whereas a closer correlation between gene expression and betacyanin content was obtained in roots of *AhIR.* It remains to be determined why better correlations were usually obtained with *AhIR*, (e.g. are fundamental differences between *AhNut* and *AhIR* due to the semi-domestication of *AhNut* for grain production?), and whether betacyanin biosynthesis pathways vary depending on the stress applied, e.g. different dependence on DOT activity, which may not be required under certain circumstances (e.g. water stress) if DOPA is converted directly to betalamic acid via DODA or to cyclo-DOPA via the Cyt P450 encoded by the *CYP76* gene [15]. However, the latter proposal is weakened by the lack of gene expression patterns showing differential induction of the above genes under water and salt stress conditions, respectively. On the other hand, it was evident that lack of pigmentation in water- and salt-stressed *AhIG* did not only coincide with mostly down-regulated DOT activity levels but with an irregular pattern of gene expression involving the down-regulation of some genes (i.e. *AhDODA-1* and *AhB5-GT*) and the late or transient expression of others. It may be relevant to add that differences in pigmentation intensity were not associated with chlorophyll loss, as observed in red leaves of *A. tricolor* [63] (results not shown).

The herbivory experiments also showed that pigment accumulation and betacyanin biosynthetic genes and enzymes were induced differentially in response to insect folivory. These results complement previous data suggesting that betacyanins may be also involved in resistance to biotic stress, as shown by: i) the participation of a ROS-inducible glucosyltransferase from beet root involved in betalain synthesis in response to wounding, bacterial infiltration and exposure to H_2_O_2_ [45], [64]; ii) an *in silico* promoter analysis of two *DODA* genes from *P*. *americana* that identified putative MYB, bHLH, and WRKY transcription factor binding sites that could regulate *PaDODA*s and betacyanin biosynthesis in response to various stresses including pathogen infection [54], and iii) data showing that methyl jasmonate and ethylene, which are elicitors associated with wounding and insect herbivory, increased betacyanin content in *A. mangostanus* seedlings [65]. However, data generated in our laboratory indicated that, contrary to the above, the defensive function of betacyanin-biosynthetic genes in amaranth might be directed against herbivores, as suggested by their observed unresponsiveness to bacterial infection (results not shown).

Although genotype factors affecting pigment, enzyme and gene expression levels were also found to influence betacyanin biosynthesis in the herbivory stress assays, they nevertheless showed that insect presence is an additional factor for the local induction of betacyanin accumulation in leaves. This was consistent with the increased DOT activity levels and the strong induction of all genes examined except *AhDODA-1* and *AhCYP76* in leaves of *AhNut* plants subjected to DH, all of which correlated with pigment accumulation. The local response was much weaker once larvae were removed from the leaves in the CH assays, meaning perhaps that an insect-derived factor produced during insect feeding was important for the local induction of an orchestrated response leading to pigment accumulation in a genotype-dependent manner. Differences between genotypes became again evident in the systemic response in plants subjected to DH, with betacyanin accumulation occurring only in roots of *AhIR*, despite the fact that betacyanin gene expression and DOT activity were also up-regulated in roots of *AhNUT*. On the other hand, the predominant expression of *AhcDOPA5-GT* in roots of *AhIR* subjected to DH could indicate that in these tissues the glucosylation at the cDOPA step is the preferred biosynthetic pathway over that involving glucosylation at the betanidin stage. Although this is a controversial aspect of betalain synthesis, it is still considered to be the preferred biosynthetic route in species closely related to amaranth (see above; [18]). However, its validation in amaranth will require further experimentation, at least in roots of amaranth plants subjected to insect herbivory. Conversely, the stronger systemic induction of betacyanin biosynthetic genes and DOT activity, which mirrored betacyanin accumulation, particularly in roots of *AhIR* plants subjected to CH could have meant that systemic signaling was being repressed to a certain degree by insect feeding. Only a few cases of suppression of defense responses produced by elicitors derived from the insects oral secretions are known [66], [67]. Moreover, the role of oral secretions in the defense response in roots is still unresolved [68]. Therefore, the biological implications of the possible manipulation of local and systemic betacyanin accumulation by insect herbivory in amaranth will require further experimentation to be determined.

A noticeable difference between the DH and CH assays and the other two stresses tested was that all three genotypes had a rather similar gene induction expression pattern in certain tissues and herbivory modalities. This suggested that the genes examined might have functions other than betacyanin biosynthesis in amaranth plants challenged by insect herbivory, especially in the acyanic *AhIG* genotype. Such possibility is supported by findings in non-Caryophyllales solanaceae plants showing that induced DODA-like proteins may be involved in other aspects of plant metabolism, including basal resistance to pathogens, nematodes, insects, cold, heat stress and wounding, possibly through the metabolism of aromatic compounds contributing to antimicrobial or antioxidative activity [69]. Moreover, studies performed with a purified betanidin glucosyltransferase recently isolated from *A. tricolor* revealed that the enzyme catalyzed the glucosylation of flavonoids (e.g. kaempferol and quercetin) in addition to its glucosylation role in betacyanin biosynthesis [70].

## Conclusions

This work reports the isolation of cDNA sequences coding for key genes in the biosynthesis of betacyanins in *A. hypochondriacus*. The phylogenetic analysis of the predicted proteins generated the expected clustering with similar proteins reported in other related betacyanin producing plants. The only exception was the AhDODA-2 protein which was more similar to DODA-like proteins found in anthocyanin producing non-Caryophyllales species. This finding might have evolutionary and physiological implications. Also relevant were the results indicating that pigment accumulation did not always correlate with betacyanin biosynthetic gene expression levels and/or DOT activity. The discrepancy observed was found to be genotype-dependent and tissue-specific and was influenced also by the type of stress applied. Also, insect-herbivory induced high levels of expression of betacyanin biosynthetic genes, which did not always coincide with pigmentation, particularly in acyanic plants. Such finding suggests that they might have functions other than betacyanin biosynthesis in *A. hypochondriacus*, probably in local and systemic defense against herbivores.

## Methods

### Plant material, insects and treatments

Seeds of *Amaranthus hypochondriacus* L cv. Nutrisol, having betacyanic tissues (origin: México), and of accessions PI 480569 (IC-38040, having betacyanic stems and roots; origin: India) and PI 481336 (IC-42287-14, with all tissues acyanic or non-pigmented; origin: India) were kindly provided by E. Espitia (INIFAP, México) and D. Brenner (USDA, Iowa State University, Ames, IA, USA), respectively. Seeds were germinated in a 60 cell black polystyrene germination seedling tray using a germination mixture consisting of 1 part Sunshine Mix 3 (SunGro Horticulture, Bellevue, WA) and 1 part coconut paste (Hummert de México, Morelos, México). Two-week-old seedlings were subsequently transplanted into 1.3-L plastic pots containing a sterile soil mixture (3 parts Sunshine Mix 3, 1 part loam, 2 parts mulch, 1 part vermiculite [SunGro Horticulture] and 1 part perlite [Termolita S.A., Nuevo León, México]) and were grown until they were 4-weeks-old and had 8 expanded leaves. Plantlets were grown for experimentation in a greenhouse under natural daylight conditions or in a growth cabinet under controlled conditions of light (≈ 300 µmol m^−2^ s^−1^) and temperature (28°C, 16 h light/8 h dark). Larvae of the lepidopteran Hawaiian beet webworm (*Spoladea recurvalis*) employed for the herbivory experiments, were taken from a colony reared in the laboratory which was started with adult specimens collected from experimental amaranth fields established nearby.

Salt- and drought-stress treatments were performed in the growth cabinets under the above conditions using six plants per genotype in each of the two biological replicates of the experiments. For salinity treatments, the plants were subjected to acute salt stress by watering the 1.3-L pots for 4 straight days with 50 mL of a 400 mM NaCl solution (electrical conductivity, in deci-Siemens per metre [dS/m], ≈20 dS/m). Soil salinity was estimated both by measuring the electrical conductivity in the excess run-off water flowing from the pots, and in the substrate in which the plants were grown, using the rapid field test for soil salinity as described in the Salinity Fact Sheet (Primary Industries and Regions South Australia [PIRSA], www.pir.sa.gov.au). The mean salinity in the run-off water sampled from the totality of the experimental pots (n  = 18), measured on the last day of the experiment, was 9.72 dS/m. Salinity in the run-off water sampled from control pots containing only soil was 12.54 dS/m. The addition of salt had no effect on the water pH, which remained stable at 7.5. On the other hand, the mean soil salinity values, as determined by the above method, were the following (in dS/m): 0.28 (in controls irrigated with water only); 3.45 (in control pots containing no plants) and 2.90 (in experimental pots). The latter values, as determined by the interpretation chart for the rapid field salinity test, represent high to severe salinity levels that affect salt-tolerant plants or are only suitable for highly salt tolerant plants.

Drought stress, without acclimation (designed to measure basal tolerance, in contrast to acquired tolerance), was imposed by withholding irrigation for 18 d (experiment 1), and 23 d (experiment 2). The water potential of the soil at the end of the drought stress treatments was −1.95 MPa and −2.5 MPa for experiments 1 and 2, respectively. These values, which were within the water-stress to drought range, were equivalent to moisture losses by the soil substrate of 93.1% and 96%, respectively. The mean relative water content in leaves of drought-stressed plants, as determined in [71] ranged from 52 to 48%, respectively. The totality of the leaves, and complete stem and root samples were collected at the end of the salt- and drought-stress experiments. The plant samples were stored at −80°C until required for further analysis.

Two different insect herbivory experiments were performed. In both, the feeding caterpillars were confined to the plants by enclosing the entire pot within plastic mesh sleeves closed at the top with a clothespin. To control for potential handling and shading effects, undamaged control plants were also enclosed within plastic mesh sleeves at the time of the herbivory treatments. In the first modality, denominated *continuous* herbivory experiments, groups of six plants were subjected to insect defoliation by allowing intermediate instar larvae of *S. recurvalis* (3 caterpillars per plant) to feed freely on the foliage for 18 h before their removal from the plants. Then, successive groups of plants were sampled for tissues (damaged leaves, stems and roots) at 1, 6, 12, 24, 48 and 72 h after the insects were removed. In the second modality, denominated *discontinuous* herbivory experiments, plant tissues from herbivore-damaged plant groups were sampled, as above, after 3, 6, 9, 12, 15 and 18 h periods of herbivory. Similarly to the above, damaged leaves, stems and roots were collected at each time point. Plant tissue samples from both experimental modalities were frozen in liquid N_2_ and stored at −80°C until required for analysis. Plant tissues of undamaged controls for each time point in both experimental modalities were also sampled. These two experimental modalities were tested considering that preliminary trials had shown that the expression of some amaranth defense-related genes can be repressed when the larvae are feeding on the plant and only resurge after insect feeding ceases. Two replicate trials of each treatment were simultaneously performed using a randomized complete block design. All experiments were performed under greenhouse conditions in the spring of 2011.

### Isolation and analysis of betacyanins

Leaf, stem and root tissue samples of the three genotypes of *A. hypochondriacus* plants subjected to the different stress treatments described above, together with those obtained from the respective controls, were homogenized in liquid nitrogen. Betacyanins were extracted in water and the pigment content in the solutions was determined by spectrophotometrical determination at 536 nm, using an Ultramark Microplate Imaging System (Bio-Rad Laboratories, Hercules CA, USA). The betacyanin content of the plant aqueous extracts was estimated using the molar extinction coefficient for amaranthine (5.66×10^4 ^L mol^−1^ cm^−1^;[22]) and a MW of 726.6.

### DOPA oxidation activity of tyrosinase: extraction and in vitro assay

DOPA oxidation activity of tyrosinase (EC 1.10.3.1; DOT) is deemed to be catalyzed by the same copper-containing bi-functional enzyme involved in the hydroxylation of tyrosine (EC 1.14.18.1) [23] The preparation of plant extracts for the determination of DOT activity was performed according to a previously described method [29] with some modifications. Briefly, leaves of *A. hypochondriacus* were ground in liquid nitrogen. Two grams of the resulting powder were re-suspended in 3.5 mL of 60 mM KPi buffer (KH_2_PO_4_/K_2_HPO_4_, pH 5.7), including 0.5 M NaCl, 10 mM ascorbic acid, 10 µM CuCl_2_ and 2% w/v polyvinylpolypyrrolidone. After the mixtures had been stirred for 15 min at 4°C, they were filtered through four layers of cheesecloth and centrifuged (15 min at 10,000 rpm/4°C). The supernatants were concentrated by ammonium sulfate precipitation (at 80% saturation) and centrifuged at 12,000 rpm and 4°C for 10 min. The pellets were re-suspended in 20 mM KPi buffer (pH 5.7) containing 10 µM CuCl_2_ and were desalted in a dialysis buffer (5 mM KPi, pH 5.7, 0.5 mM ascorbic acid) using a Spectra/Por membrane (Spectrum Laboratories, Rancho Dominguez, CA, USA). The protein content of the dialyzed extracts was determined using the Bradford dye-binding method [72], using human serum albumin as a standard. Enzyme extracts were stored at −80°C until analysis.

The *in vitro* assays of DOT activity were performed according to reported methods [29], [30] with some modifications. The reaction mixture (250 µL) contained 125 µL of assay buffer (120 mM KPi [pH 6.8]), 4% *N,N*-dimethylformamide, 44 µL of 28.6 mM 3-methyl-2-benzothiazolinone hydrazone hydrochloride hydrate (MBTH), 75 µL of 10 mM L-DOPA and 6.3 µL of enzyme extract. The assay buffer and the MBTH stock solution were saturated with O_2_ for 20 min immediately before use. The buffer, MBTH solution, and enzyme extracts were pre-incubated at room temperature for 1 min before the reaction was started by the addition of the substrate. The increase of absorbance at 490 nm was monitored in 30 s intervals over a period of 1 min and in 60 s intervals over 10 min with a spectrophotometer (Bio-Rad). DOT activity was reported as Δ OD (at 490 nm)/min/mg protein. The blank was measured with buffer instead of enzyme preparation. Positive controls were run using mushroom (*Agaricus bisporus*) tyrosinase (Sigma-Aldrich Co., St. Louis, MO, USA). All assays were performed in triplicate.

### RNA isolation and cDNA preparation

Total RNA was extracted from 100–200 mg of frozen tissue with the Trizol reagent (Invitrogen, Carlsbad, CA, USA), according to the manufactureŕs instructions with modifications. These consisted of the addition of a salt solution (sodium citrate 0.8 M+1.2 M NaCl) during precipitation in a 1∶1 v/v ratio with isopropanol and further purification with LiCl (8 M) for 1 h at 4°C. All RNA samples were analyzed by formaldehyde agarose gel electrophoresis and visual inspection of the ethidium bromide-stained ribosomal RNA bands. Total RNA samples (1 µg) were reverse-transcribed to generate the cDNA first-strands using an oligo dT_20_ primer and 200 units of SuperScript II reverse transcriptase (Invitrogen).

### Full-length cDNA amplification

In order to amplify full-length *AhDODA-1*, *AhDODA-2*, *AhcDOPA5-GT* and *AhB5-GT* cDNAs, total RNA samples (1 µg) from leaves of grain amaranth plantlets were reverse-transcribed to generate the cDNA first-strands as described above. An aliquot of these reactions (2 µl) was then directly used as template in all PCR reactions in the presence of 100 pmol each of specific primers (for *AhDODA-1*, *AhDODA-2*, and *AhcDOPA5-GT*) designed on the basis of sequences obtained from the *Ah* transcriptome [73] or of degenerate primers designed on the basis of an amino acid consensus sequence derived from a multiple alignment of glycosyltransferases from *Beta vulgaris* (accession numbers AY240951 and AY526080), *Dianthus caryophyllus* (accession number AB191248), *Dorotheanthus bellidiformis* (accession number Y18871), *Nicotiana tabacum* (accession number U32643), *Phytolacca americana* (accession number AB458517) and *Solanum lycopersicum* (accession number X85138). The alignment was constructed with ClustalW2 Multiple Sequence Alignment (EMBL-EBI, Wellcome Trust Genome Campus, Hinxton, Cambridgeshire, UK). RACE primers for all sequences were designed from the available partial cDNA sequences using Primer3Plus software (http://www.bioinformatics.nl/cgi-bin/primer3plus/primer3plus.cgi) (File S4). The amplification of the 5′ and 3′ cDNA ends was carried out by RACE (Rapid Amplification of cDNA Ends) with the SMARTer RACE cDNA Amplification Kit (Clontech, Laboratories, Mountain View, CA, USA), according to the manufacturer's instructions. The fragments obtained were cloned in pCR 4.0 TOPO vectors (Invitrogen) and were subsequently sequenced to confirm that they corresponded to the gene of interest. All complete cDNA sequences were deposited in the GenBank as HQ889614 (*AhDODA-1*), KJ136016 (*AhDODA-2*), KJ136017 (*AhB5-GT*), KJ136018 (*AhcDOPA5-GT*), and KJ136019 (partial *AhCYP76*).

### Construction of phylogenetic trees

Complete (AhDODA-1, AhDODA-2, AhcDOPA5-GT and AhB5-GT) and partial (AhCYP76) deduced amino acid sequences of the *A. hypochondriacus* betacyanin-biosynthetic enzymes were used as a query to obtain homology with the predicted amino acid sequences from other species identified from GenBank using BLAST (National Center for Biotechnology Information, NCBI). Results were selected with a total score >200 (E value < 1e^−59^). Multiple sequence alignment was performed using Clustal W Multiple Alignment program [74]. Phylogenetic and molecular evolutionary analyses were conducted with *MEGA* version 5 [75], using the Neighbor-Joining algorithm. The accession numbers of the sequences used to construct the phylogenetic tree are included in the respective tree-outputs.

### Gene expression analysis by quantitative real-time RT-PCR (qRT-PCR)

The cDNA employed for the qRT-PCR assays was initially prepared from 4 µg total RNA. It was then diluted 10-fold in sterile deionized-distilled (dd) water prior to qRT-PCR. Amplifications were performed using SYBR Green detection chemistry and run in triplicate in 96-well reaction plates with the CFX96 Real Time System (Bio-Rad). Reactions were prepared in a total volume of 20 µL containing: 2 µL of template, 2 µL of each amplification primer (2 mM), 8 µL of IQ SYBR SuperMix (Bio-Rad) and 6 µL of sterile dd water. Quantitative real-time PCR was performed in triplicate for each sample using the primers listed in File S5. Primers were designed for each gene, based on partial cDNA sequences derived from the transcriptomic analysis of *Ah* (i.e. *AhCYP76*) [73] or from complete cDNAs generated in this study (see above). Primer design was performed using DNA calculator software (Sigma-Aldrich) and included, when possible, part of unique 3′ non-coding regions to ensure specificity.

The following protocol was followed for all qRT-PCR runs: 15 min at 95°C to activate the *Taq* Polymerase, followed by 40 cycles of denaturation at 95°C for 15 s and annealing at 60°C for 1 min. All amplifications requiring an excess of 32 cycles were not considered for analysis. The specificity of the amplicons was verified by melting curve analysis after 40 cycles and agarose gel electrophoresis. Baseline and threshold cycles (Ct) were automatically determined using Real-Time PCR System software. PCR efficiencies for all genes tested were greater than 95%. Relative expression was calculated using the comparative cycle threshold method [76], where delta (Δ) cycle threshold of cDNA from undamaged controls was defined as 100% transcript presence.

Transcript abundance data were normalized against the average transcript abundance of two reference genes: actin (isotig 10321) and β-tubulin (isotig 05486). These were obtained from the above transcriptomic study. The fold change in expression of the target genes in each treatment was calculated using the following equation: 2^−ΔΔCt^, where ΔΔCt  =  (Ct target gene - average Ct reference genes)_treatment_ - (Ct target gene - average Ct reference genes)_control_. Values reported are the mean of three repetitions ± SE of one representative experiment.

The cDNA samples were tested by using three independent repetitions in the same condition. Each RNA sample was isolated from leaves, stems and roots of six individual plants for each cultivar and treatment and pooled to produce one final sample. Data are presented as mean ± SE of three technical replicates of the pooled samples. The qRT-PCR expression analysis of the betacyanin-biosynthetic genes included in this study, was validated in two independent experiments. The relative expression of target genes to the actin and tubulin controls was calculated using the efficiency-adjusted ΔCt method as described in Yuan [77].

### Statistical analysis

All statistical analyses were done using JMP 8.0.2 (SAS Institute Inc.; SAS Campus Drive, Cary, NC, USA). Data were analyzed using an ANOVA. Tukey and Dunnett tests were performed at the α = 0.05 level.

## Supporting Information

File S1 Comparison of deduced amino acid sequences of plant 4, 5-DOPA-extradiol-dioxygenases.
(PDF)

File S2 Comparison of deduced amino acid sequences of plant cyclo-DOPA 5-glycosyl-transferases.
(PDF)

File S3 Comparison of deduced amino acid sequences of plant betanidin 5-glycosyl-transferases.
(PDF)

File S4 Primers used to amplify the 5' and 3' cDNA ends (RACE) of betacyanin biosynthetic genes.
(DOCX)

File S5 Primers used for gene expression analysis by qRT PCR.
(DOCX)

## References

[1] Huerta-Ocampo JA, Barba de la Rosa AP (2011) Amaranth: a pseudo-cereal with nutraceutical properties. Curr Nutr Food Sci 7: 1–9.

[2] Venskutonis PR, Kraujalis P (2013) Nutritional components of amaranth seeds and vegetables: a review on composition, properties, and uses. Compr Rev Food Sci F 12: 381–412. 10.1111/1541-4337.12021 33412681

[3] Brenner DM, Baltensperger DD, Kulakow PA, Lehmann JW, Myers RL, et al (2000) Genetic resources and breeding of *Amaranthus* . Plant Breed Rev 19: 227–285.

[4] Myers R (1996) Amaranth: New crop opportunity. In: Janick J, editor. Progress in new crops. Alexandria, VA: ASHS Press. pp. 207–220.

[5] Omami EN, Hammes PS, Robbertse PJ (2006) Differences in salinity tolerance for growth and water-use efficiency in some amaranth (*Amaranthus* spp.) genotypes. New Zeal J Crop Hort 34: 11–22.

[6] Weber LE (1990) Amaranth grain production guide. Rodale Press, Emmaus, PA.

[7] Hauptli H (1977) Agronomic potential and breeding strategy for grain amaranths. In Proc Amaranth Seminar, 1st. Maxatawny, PA: Emmaus, PA.

[8] Huerta-Ocampo JA, Briones-Cerecero EP, Mendoza-Hernandez G, De Leon-Rodriguez A, Barba de la Rosa AP (2009) Proteomic analysis of amaranth (*Amaranthus hypochondriacus* L.) leaves under drought stress. Int J Plant Sci 170: 990–998.

[9] Huerta-Ocampo JA, Leon-Galvan MF, Ortega-Cruz LB, Barrera-Pacheco A, De Leon-Rodriguez A, et al (2011) Water stress induces up-regulation of DOF1 and MIF1 transcription factors and down-regulation of proteins involved in secondary metabolism in amaranth roots (*Amaranthus hypochondriacus* L.). Plant Biol 13: 472–482. 21489098 10.1111/j.1438-8677.2010.00391.x

[10] Johnson BL, Henderson TL (2002) Water use patterns of grain amaranth in the Northern Great Plains. Agron J 94: 1437–1443.

[11] Piha MI (1995) Yield potential, fertility requirements, and drought tolerance of grain amaranth compared with maize under Zimbabwean conditions. Trop Agr 72: 7–12.

[12] Putnam DH (1990) Agronomic practices for grain amaranth. Proc Natl Amaranth Symp, 4th. Minnesota Ext. Serv. University of Minnesota, St Paul, MN. USA. pp. 151–162.

[13] Brockington SF, Walker RH, Glover BJ, Soltis PS, Soltis DE (2011) Complex pigment evolution in the Caryophyllales. New Phytol 190: 854–864. 21714182 10.1111/j.1469-8137.2011.03687.x

[14] Han XH, Gao ZJ, Xiao XG (2009) Enzymes and genes involved in the betalain biosynthesis in higher plants. Afr J Biotechnol 8: 6735–6744.

[15] Gandia-Herrero F, Garcia-Carmona F (2013) Biosynthesis of betalains: yellow and violet plant pigments. Trends Plant Sci 18: 334–343. 23395307 10.1016/j.tplants.2013.01.003

[16] Cai YZ, Sun M, Corke H (2001) Identification and distribution of simple and acylated betacyanins in the Amaranthaceae. J Agr Food Chem 49: 1971–1978. 11308355 10.1021/jf000963h

[17] Strack D, Vogt T, Schliemann W (2003) Recent advances in betalain research. Phytochemistry 62: 247–269. 12620337 10.1016/s0031-9422(02)00564-2

[18] Tanaka Y, Sasaki N, Ohmiya A (2008) Biosynthesis of plant pigments: anthocyanins, betalains and carotenoids. Plant J 54: 733–749. 18476875 10.1111/j.1365-313X.2008.03447.x

[19] Cai YZ, Sun M, Wu HX, Huang RH, Corke H (1998) Characterization and quantification of betacyanin pigments from diverse *Amaranthus* species. J Agr Food Chem 46: 2063–2070.

[20] Gins MS, Gins VK, Kononkov PF (2002) Change in the biochemical composition of amaranth leaves during selection for increased amaranthine content. Appl Biochem Microbiol 38: 474–479. 12391759

[21] Piatelli M, Minale L (1966) Structure of amaranthine and isoamaranthine. Ann Chim (Rome) 56: 1060.

[22] Piattelli M, Giudici de Nicola M, Castrogiovanni V (1969) Photocontrol of amaranthin synthesis in *Amaranthus tricolor* . Phytochemistry 8: 731–736.

[23] Pavoković D, Krsnik-Rasol M (2011) Complex biochemistry and biotechnological production of betalains. Food Technol Biotech 49: 145–155.

[24] Gandia-Herrero F, Escribano J, Garcia-Carmona F (2007) Characterization of the activity of tyrosinase on betanidin. J Agr Food Chem 55: 1546–1551. 17256962 10.1021/jf062858z

[25] Gandia-Herrero F, Garcia-Carmona F, Escribano J (2004) Purification and characterization of a latent polyphenol oxidase from beet root (*Beta vulgaris* L.). J Agr Food Chem 52: 609–615. 14759157 10.1021/jf034381m

[26] Hirano H, Komamine A (1994) Correlation of betacyanin synthesis with cell-division in cell-suspension cultures of *Phytolacca americana* . Physiol Plant 90: 239–245.

[27] Joy RW IV, Sugiyama M, Fukuda H, Komamine A (1995) Cloning and characterization of polyphenol oxidase cDNAs of *Phytolacca americana* . Plant Physiol 107: 1083–1089. 7539531 10.1104/pp.107.4.1083 PMC157240

[28] Sakuta M, Takagi T, Komamine A (1986) Growth related accumulation of betacyanin in suspension cultures of *Phytolacca americana* . J Plant Physiol 125: 337–343.

[29] Steiner U, Schliemann W, Bohm H, Strack D (1999) Tyrosinase involved in betalain biosynthesis of higher plants. Planta 208: 114–124.

[30] Wang CQ, Song H, Gong XZ, Hu QG, Liu F, et al (2007) Correlation of tyrosinase activity and betacyanin biosynthesis induced by dark in C3 halophyte *Suaeda salsa* seedlings. Plant Sci 173: 487–494.

[31] Yamamoto K, Kobayashi N, Yoshitama K, Teramoto S, Komamine A (2001) Isolation and purification of tyrosine hydroxylase from callus cultures of *Portulaca grandiflora* . Plant Cell Physiol 42: 969–975. 11577191 10.1093/pcp/pce125

[32] Hatlestad GJ, Sunnadeniya RM, Akhavan NA, Gonzalez A, Goldman IL, et al (2012) The beet R locus encodes a new cytochrome P450 required for red betalain production. Nat Genet 44: 816–820. 22660548 10.1038/ng.2297

[33] Hempel J, Bohm H (1997) Betaxanthin pattern of hairy roots from *Beta vulgaris* var. *lutea* and its alteration by feeding of amino acids. Phytochemistry 44: 847–852.

[34] Schliemann W, Kobayashi N, Strack D (1999) The decisive step in betaxanthin biosynthesis is a spontaneous reaction. Plant Physiol 119: 1217–1232. 10198080 10.1104/pp.119.4.1217 PMC32006

[35] Kujala T, Loponen J, Pihlaja K (2001) Betalains and phenolics in red beetroot (*Beta vulgaris*) peel extracts: extraction and characterisation. Z Naturforsch C 56: 343–348. 11421447 10.1515/znc-2001-5-604

[36] Sasaki N, Adachi T, Koda T, Ozeki Y (2004) Detection of UDP-glucose : cyclo-DOPA 5-O-glucosyltransferase activity in four o'clocks (*Mirabilis jalapa* L.). FEBS Lett 568: 159–162. 15196939 10.1016/j.febslet.2004.04.097

[37] Sasaki N, Wada K, Koda T, Kasahara K, Adachi T, et al (2005) Isolation and characterization of cDNAs encoding an enzyme with glucosyltransferase activity for cyclo-DOPA from four o'clocks and feather cockscombs. Plant Cell Physiol 46: 666–670. 15695438 10.1093/pcp/pci064

[38] Wyler H, Meuer U, Bauer J, Stravs-Mombelli L (1984) Cyclodopa glucoside ( = (2S)-5-(β-D-glucopyranosyloxy)-6-hydroxyindoline-2-carboxylic acid) and its occurrence in red beet (*Beta vulgaris* var. *rubra* L.). Helv Chim Acta 67: 1348–1355.

[39] Sciuto S, Oriente G, Piattelli M, Impellizzeri G, Amico V (1974) Biosynthesis of amaranthin in *Celosia plumosa* . Phytochemistry 13: 947–951.

[40] Sasaki N, Abe Y, Wada K, Koda T, Goda Y, et al (2005) Amaranthin in feather cockscombs is synthesized via glucuronylation at the cyclo-DOPA glucoside step in the betacyanin biosynthetic pathway. J Plant Res 118: 439–442. 16247652 10.1007/s10265-005-0237-z

[41] Christinet L, Burdet FRX, Zaiko M, Hinz U, Zryd JP (2004) Characterization and functional identification of a novel plant 4, 5-extradiol dioxygenase involved in betalain pigment biosynthesis in *Portulaca grandiflora* . Plant Physiol 134: 265–274. 14730069 10.1104/pp.103.031914 PMC316306

[42] Mueller LA, Hinz U, Uze M, Sautter C, Zryd JP (1997) Biochemical complementation of the betalain biosynthetic pathway in *Portulaca grandiflora* by a fungal 3, 4-dihydroxyphenylalanine dioxygenase. Planta 203: 260–263.

[43] Sasaki N, Abe Y, Goda Y, Adachi T, Kasahara K, et al (2009) Detection of DOPA 4, 5-dioxygenase (DOD) activity using recombinant protein prepared from *Escherichia coli* cells harboring cDNA encoding *DOD* from *Mirabilis jalapa* . Plant Cell Physiol 50: 1012–1016. 19366710 10.1093/pcp/pcp053

[44] Moreno D, García-Viguera C, Gil J, Gil-Izquierdo A (2008) Betalains in the era of global agri-food science, technology and nutritional health. Phytochem Rev 7: 261–280.

[45] Sepúlveda-Jiménez G, Rueda-Benítez P, Porta H, Rocha-Sosa M (2004) Betacyanin synthesis in red beet (*Beta vulgaris*) leaves induced by wounding and bacterial infiltration is preceded by an oxidative burst. Physiol Mol Plant Pathol 64: 125–133.

[46] Shao L, Li Y, Wu X, Peng C (2008) Comparison on antioxidative capability in leaves of red and green amaranth (*Amaranthus tricolor* L.) under high temperature stress. Plant Physiol Commun 44: 923–926.

[47] Wang CQ, Chen M, Wang BS (2007) Betacyanin accumulation in the leaves of C_3_ halophyte *Suaeda salsa* L. is induced by watering roots with H_2_O_2_ . Plant Sci 172: 1–7.

[48] Edreva A (2005) The importance of non-photosynthetic pigments and cinnamic acid derivatives in photoprotection. Agric Ecosyst Environ 106: 135–146.

[49] Ibdah M, Krins A, Seidlitz HK, Heller W, Strack D, et al (2002) Spectral dependence of flavonol and betacyanin accumulation in *Mesembryanthemum crystallinum* under enhanced ultraviolet radiation. Plant Cell Environ 25: 1145–1154.

[50] Shao L, Chen XW, Chen YJ, Sun BY, Chow W, et al (2013) Differential responses of photosystem II activity to photooxidation in red and green tissues of *Amaranthus tricolor* leaves. Pak J Bot 45: 1905–1912.

[51] Wang CQ, Zhao JQ, Chen M, Wang BS (2006) Identification of betacyanin and effects of environmental factors on its accumulation in halophyte *Suaeda salsa* . J Plant Physiol Mol Biol 32: 195–201. 16622319

[52] Mosco A (2012) Tissue localization of betacyanins in cactus stems. Rev Mex Biodivers 83: 413–420.

[53] Massey B (2012) Understanding betalain regulation in floral and vegetative tissues of *Ptilotus* cultivars [PhD Thesis]: School of Agriculture and Food Sciences, The University of Queensland.

[54] Takahashi K, Takamura E, Sakuta M (2009) Isolation and expression analysis of two DOPA dioxygenases in *Phytolacca americana* . Z Naturforsch C 64: 564–573. 19791510 10.1515/znc-2009-7-816

[55] Zhao SZ, Sun HZ, Chen M, Wang BS (2010) Light-regulated betacyanin accumulation in euhalophyte *Suaeda salsa* calli. Plant Cell Tiss Org 102: 99–107.

[56] Kumon K, Sasaki J, Sejima M, Takeuchi Y, Hayashi Y (1990) Betacyanin decolorizing enzymes from *Phytolacca americana* . Plant Cell Physiol 31: 233–240.

[57] Martinez-Parra J, Munoz R (2001) Characterization of betacyanin oxidation catalyzed by a peroxidase from *Beta vulgaris* L. roots. J Agr Food Chem 49: 4064–4068. 11513711 10.1021/jf0013555

[58] Bowles D, Isayenkova J, Lim EK, Poppenberger B (2005) Glycosyltransferases: managers of small molecules. Curr Opin Plant Biol 8: 254–263. 15860422 10.1016/j.pbi.2005.03.007

[59] Bowles D, Lim EK, Poppenberger B, Vaistij FE (2006) Glycosyltransferases of lipophilic small molecules. Annu Rev Plant Biol 57: 567–597. 16669774 10.1146/annurev.arplant.57.032905.105429

[60] Wang CQ, Wang BS (2007) Ca^2+^-calmodulin is involved in betacyanin accumulation induced by dark in C3 halophyte *Suaeda salsa* . J Integr Plant Biol 49: 1378–1385.

[61] Georgiev VG, Weber J, Kneschke EM, Denev PN, Bley T, et al (2010) Antioxidant activity and phenolic content of betalain extracts from intact plants and hairy root cultures of the red beetroot *Beta vulgaris* cv. Detroit Dark Red. Plant Foods Hum Nutr 65: 105–111. 20195764 10.1007/s11130-010-0156-6

[62] Stagnari F, Galieni A, Speca S, Pisante M (2014) Water stress effects on growth, yield and quality traits of red beet. Sci Hortic 165: 13–22.

[63] Iwamoto K, Fukuda H, Sugiyama M (2001) Elimination of POR expression correlates with red leaf formation in *Amaranthus tricolor* . Plant J 27: 275–284. 11532173 10.1046/j.1365-313x.2001.01082.x

[64] Sepulveda-Jimenez G, Rueda-Benitez P, Porta H, Rocha-Sosa M (2005) A red beet (*Beta vulgaris*) UDP-glucosyltransferase gene induced by wounding, bacterial infiltration and oxidative stress. J Exp Bot 56: 605–611. 15582929 10.1093/jxb/eri036

[65] Cao S, Liu T, Jiang Y, He S, Harrison DK, et al (2012) The effects of host defence elicitors on betacyanin accumulation in *Amaranthus mangostanus* seedlings. Food Chem 134: 1715–1718. 23442611 10.1016/j.foodchem.2012.03.129

[66] Bos JIB, Prince D, Pitino M, Maffei ME, Win J, et al (2010) A functional genomics approach identifies candidate effectors from the aphid species *Myzus persicae* (green peach aphid). PLoS Genet 6: e1001216. 21124944 10.1371/journal.pgen.1001216 PMC2987835

[67] Eichenseer H, Mathews MC, Bi JL, Murphy JB, Felton GW (1999) Salivary glucose oxidase: multifunctional roles for *Helicoverpa zea*? Arch Insect Biochem 42: 99–109. 10.1002/(SICI)1520-6327(199909)42:1<99::AID-ARCH10>3.0.CO;2-B 10467060

[68] Fürstenberg-Hägg J, Zagrobelny M, Bak S (2013) Plant defense against insect herbivores. Int J Mol Sci 14: 10242–10297. 23681010 10.3390/ijms140510242 PMC3676838

[69] Bahramnejad B, Erickson LR, Goodwin PH (2010) Induction of expression and increased susceptibility due to silencing a 4, 5-DOPA dioxygenase extradiol-like gene of *Nicotiana benthamiana* in the interaction with the hemibiotrophic pathogens, *Colletotrichum destructivum*, *Colletotrichum orbiculare* or *Pseudomonas syringae* pv. *tabaci* . Plant Sci 178: 147–157.

[70] Das SS, Gauri SS, Misra BB, Biswas M, Dey S (2013) Purification and characterization of a betanidin glucosyltransferase from *Amaranthus tricolor* L catalyzing non-specific biotransformation of flavonoids. Plant Sci 211: 61–69. 23987812 10.1016/j.plantsci.2013.07.003

[71] Barrs HD, Weatherley PE (1962) A re-examination of relative turgidity technique for estimating water deficits in leaves. Aust J Biol Sci 15: 413–428.

[72] Bradford MM (1976) A rapid and sensitive method for quantitation of microgram quantities of protein utilizing principle of protein-dye binding. Anal Biochem 72: 248–254. 942051 10.1016/0003-2697(76)90527-3

[73] Delano-Frier JP, Aviles-Arnaut H, Casarrubias-Castillo K, Casique-Arroyo G, Castrillon-Arbelaez PA, et al (2011) Transcriptomic analysis of grain amaranth (*Amaranthus hypochondriacus)* using 454 pyrosequencing: comparison with *A. tuberculatus*, expression profiling in stems and in response to biotic and abiotic stress. BMC Genomics 12: 363. 21752295 10.1186/1471-2164-12-363 PMC3146458

[74] Thompson JD, Higgins DG, Gibson TJ (1994) CLUSTAL W: improving the sensitivity of progressive multiple sequence alignment through sequence weighting, position-specific gap penalties and weight matrix choice. Nucleic Acids Res 22: 4673–4680. 7984417 10.1093/nar/22.22.4673 PMC308517

[75] Tamura K, Peterson D, Peterson N, Stecher G, Nei M, et al (2011) MEGA5: molecular evolutionary genetics analysis using maximum likelihood, evolutionary distance, and maximum parsimony methods. Mol Biol Evol 28: 2731–2739. 21546353 10.1093/molbev/msr121 PMC3203626

[76] Livak KJ, Schmittgen TD (2001) Analysis of relative gene expression data using real-time quantitative PCR and the 2^−ΔΔC_T_^ method. Methods 25: 402–408. 11846609 10.1006/meth.2001.1262

[77] Yuan JS, Burris J, Stewart NR, Mentewab A, Stewart CN (2007) Statistical tools for transgene copy number estimation based on real-time PCR. BMC Bioinformatics 8 (Suppl 7)S6. 10.1186/1471-2105-8-S7-S6 PMC2099498 18047729

