# Supplementary figures and images for: Betacyanin Biosynthetic Genes and Enzymes Are Differentially Induced by (a)biotic Stress in Amaranthus hypochondriacus
Source: PLoS One. 2014 Jun 4;9(6):e99012. doi: 10.1371/journal.pone.0099012 (PMC4045864; doi:10.1371/journal.pone.0099012)

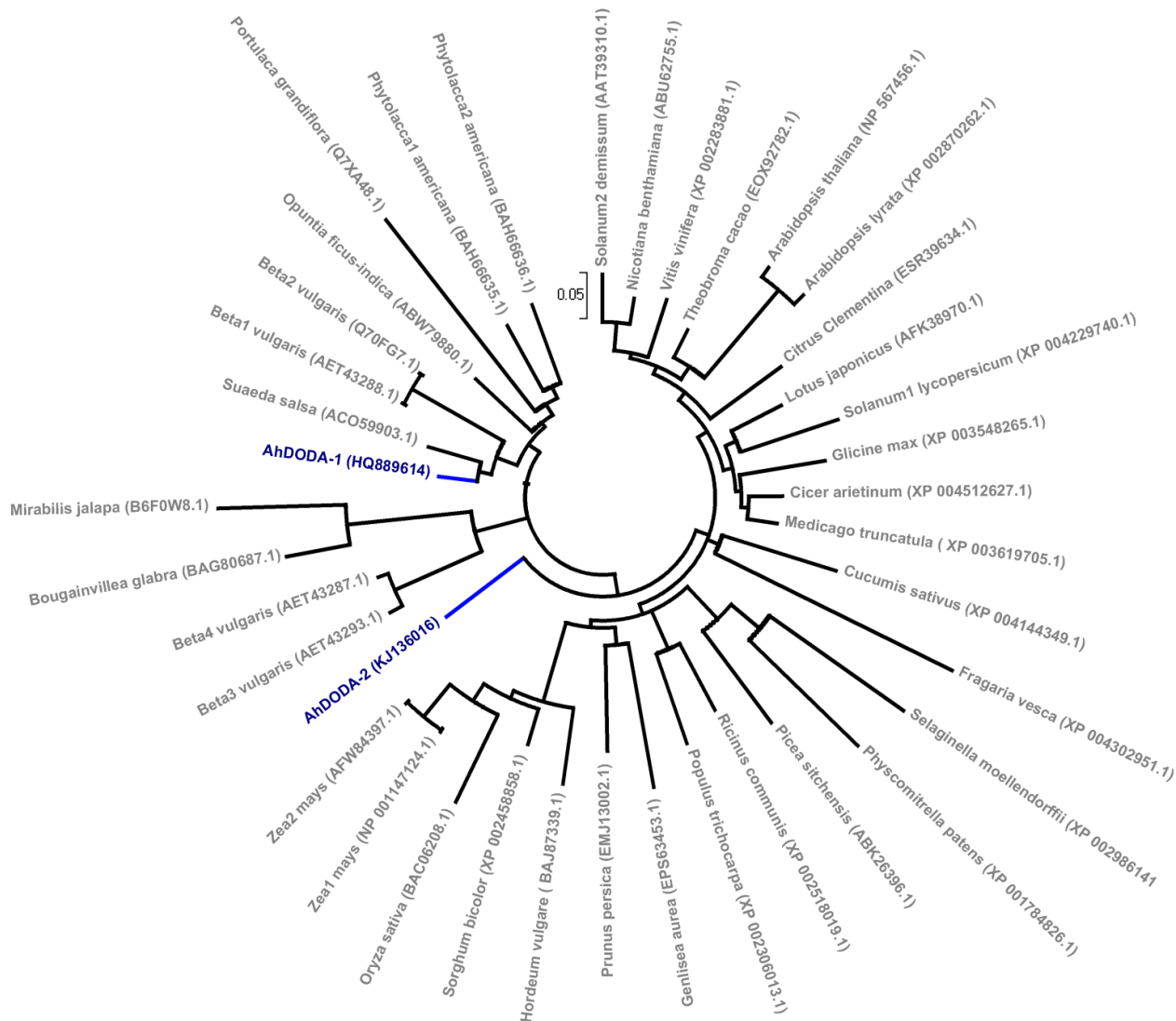

**File S1: Comparison of deduced amino acid sequences of plant 4, 5-DOPA-extradiol dioxygenases.**

Supplement: File S1 — Comparison of deduced amino acid sequences of plant 4, 5-DOPA-extradiol-dioxygenases. (PDF) [file pone.0099012.s001.pdf]

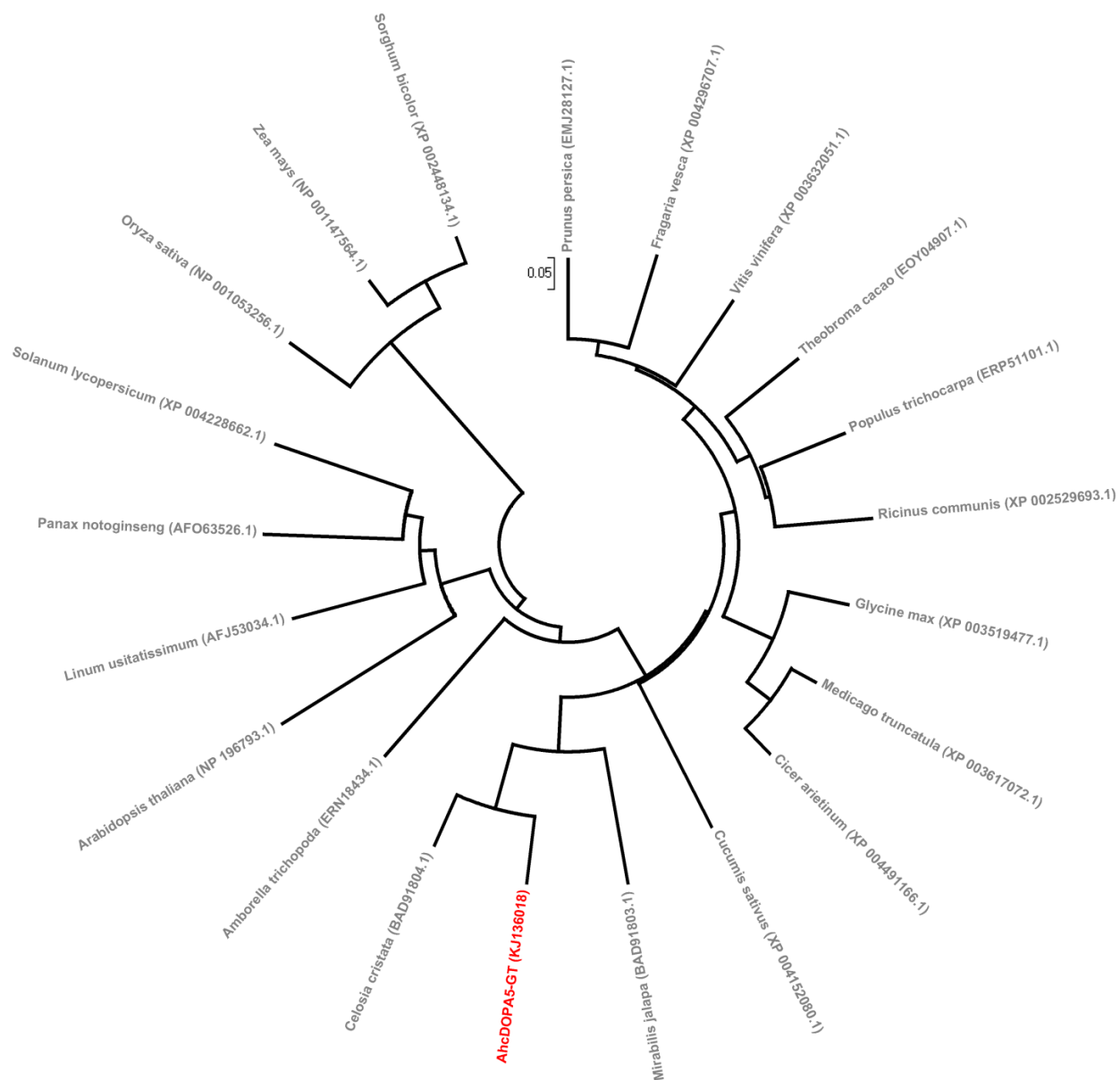

**File S2: Comparison of deduced amino acid sequences of plant cyclo-DOPA 5-glycosyl-transferases .**

Supplement: File S2 — Comparison of deduced amino acid sequences of plant cyclo-DOPA 5-glycosyl-transferases. (PDF) [file pone.0099012.s002.pdf]

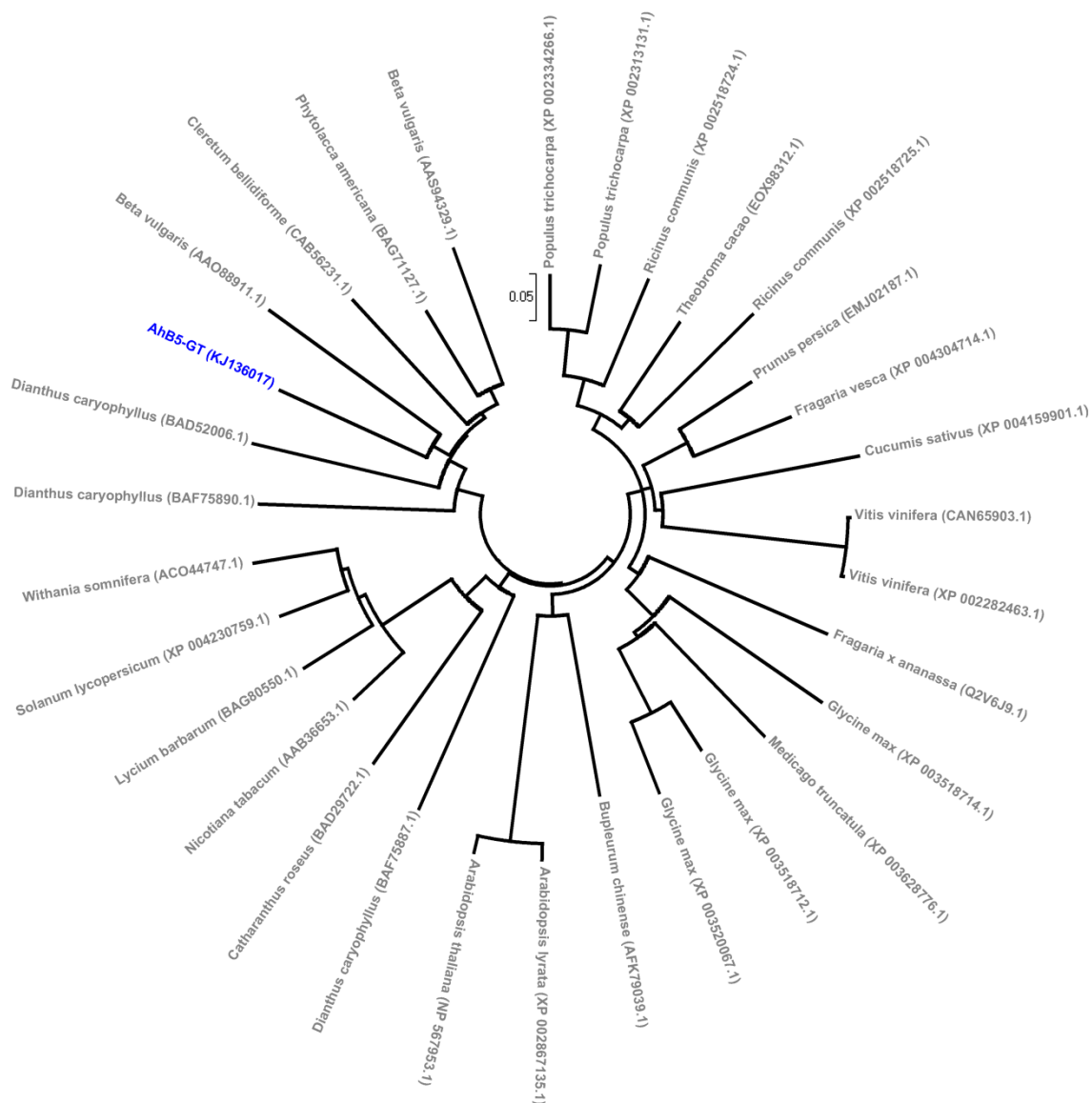

**File S3: Comparison of deduced amino acid sequences of plant betanidin 5-glycosyl-transferases .**

Supplement: File S3 — Comparison of deduced amino acid sequences of plant betanidin 5-glycosyl-transferases. (PDF) [file pone.0099012.s003.pdf]
