# Supplementary material for: Betacyanin Biosynthetic Genes and Enzymes Are Differentially Induced by (a)biotic Stress in Amaranthus hypochondriacus
Source: PLoS One. 2014 Jun 4;9(6):e99012. doi: 10.1371/journal.pone.0099012 (PMC4045864; doi:10.1371/journal.pone.0099012)
Supplement: File S4 — Primers used to amplify the 5' and 3' cDNA ends (RACE) of betacyanin biosynthetic genes. (DOCX) [file pone.0099012.s004.docx]

File S4. Primers used for RACE

| Gene |  | | Sequence | |
| --- | --- | --- | --- | --- |
| *Betanidin 5-glycosyl transferase* (*AhB5-GT*) | | Race 5’ | | AATTGGGAAGGAGAGCATGGCATATTG |
|  |  | Race 3’ | | GTTAAGCCATTTTAAGCACTCGTGTTCATC |
|  |  |  | |  |
| *4, 5-DOPA-extradiol-dioxygenase* (*Ah DODA-1*) | | Race 5’ | | TGGCTTGAAACTGCCCTGACAAATGG |
|  |  | Race 3’ | | CTCCAGCGGCTCCCATTGCTACAT |
|  |  |  | |  |
| *cyclo-DOPA 5-glycosyl-transferase* (*AhcDOPA5GT)* | | Race 5’ | | CCACGCAGAACAATTCCTGGAGTTCTT |
|  |  |  | |  |
| *4, 5-DOPA-extradiol-dioxygenase* (*Ah DODA-1*) | | Race 5’ | | CTGGTGATGGATGAGCCTTTTTTGC |
