# Supplementary material for: Betacyanin Biosynthetic Genes and Enzymes Are Differentially Induced by (a)biotic Stress in Amaranthus hypochondriacus
Source: PLoS One. 2014 Jun 4;9(6):e99012. doi: 10.1371/journal.pone.0099012 (PMC4045864; doi:10.1371/journal.pone.0099012)
Supplement: File S5 — Primers used for gene expression analysis by qRT PCR. (DOCX) [file pone.0099012.s005.docx]

File S5. Primers used for gene expression analysis by qRT PCR.

| Gene |  | Sequence |
| --- | --- | --- |
| *cyclo-DOPA 5-glycosyl-transferase* (*AhcDOPA5-GT*) | Forward | GAAATTGAGGGATGCTATGAAAGT |
|  | Reverse | CTTCAATTTTGGTGCTAGCTCA |
|  |  |  |
| *4, 5-DOPA-extradiol-dioxygenase 1* (*AhDODA-1*) | Forward | GTCTTATGGATCCTACAAATTTG |
|  | Reverse | AACACAACAATAATAAACAAACAGTAGA |
|  |  |  |
| *4, 5-DOPA-extradiol-dioxygenase* *2* (*AhDODA-2)* | Forward | TATGCCTCCTACAAGTTCACTG |
|  | Reverse | CTAAACAACACAACATGCATCTTAT |
|  |  |  |
| *Betanidin 5-glycosyl transferase* (*AhB5-GT*) | Forward | TGTTGAAGTGGATGGATCTTCATATAA |
|  | Reverse | TTGCAGGATTCTTAGTTTTGATATCAC |
|  |  |  |
| *Cytochrome P-450 R gene* (*AhCYP76*) | Forward | GTCATGGTTACCCGTTTCAC |
|  | Reverse | TGCAGCTTTACCGATATCAA |
|  |  |  |
| Actin (*AhATC*) | Forward | CGTGACCTGACTGATTACCTTA |
|  | Reverse | GCTCGTAGTTCTTCTCAATGGC |
|  |  |  |
| β-tubulin (*AhTUB*) | Forward | TCTCAGCAGTATGTCTCCCTCA |
|  | Reverse | TCTACTTCTTTGGTGCTCATCTT |
